# Correction to: The rumen microbiome as a reservoir of antimicrobial resistance and pathogenicity genes is directly affected by diet in beef cattle

**DOI:** 10.1186/s40168-019-0764-9

**Published:** 2019-11-18

**Authors:** Marc D. Auffret, Richard J. Dewhurst, Carol-Anne Duthie, John A. Rooke, R. John Wallace, Tom C. Freeman, Robert Stewart, Mick Watson, Rainer Roehe

**Affiliations:** 1 SRUC, Edinburgh, EH25 9RG UK; 2 Rowett Institute, University of Aberdeen, Aberdeen, AB25 2ZD UK; 3 Division of Genetics and Genomics, The Roslin Institute and R(D) SVS, University of Edinburgh, Edinburgh, EH25 9RG UK; 4 Edinburgh Genomics, The Roslin Institute and R(D)SVS, University of Edinburgh, Edinburgh, EH25 9RG UK

**Correction to: Microbiome (2017) 5: 159**

**https://doi.org/10.1186/s40168-017-0378-z**

Following publication of the original article [1], the authors reported an error in the Additional file 1. The revised manuscript was mistakenly uploaded as the Additional file 1. The correct file that includes Supplementary Figures and Tables is available here.

The publishers apologise for this error.

## Supplementary information

**Additional file 1: Figure S1.**Relative abundance (%) of 20 groups of functional genes representing 204 selected genes (number of animals, *n* = 50 samples). The sum of the relative abundance (%) of genes grouping within the same function is shown in this figure. **Figure S2A.** Total abundance of 204 selected genes based on diet treatments (*n* = 50). *P value < 0.05. **Figure S2B.** Shannon index diversity of 204 selected genes based on diet treatments (*n* = 50). *P value < 0.05, °*P* value < 0.1. **Figure S3.** Canonical Variate analysis (CVA) on the structure of 204 genes selected based on breed, age, weight, *Proteobacteria* ratio, FCR and methane grouping (*n* = 50). **Figure S4.** Factors explaining the significant differences observed for *Proteobacteria* ratio (*n* = 50). **Figure S5.** Microbial community composition at the phylum level (*n* = 50). **Table S1**. Characteristics of the cattle used in the experiment. **Table S2.** Groups of AMR genes significantly correlated with abundance of the *Proteobacteria* phylum and *Proteobacteria* ratio. **Table S3.** The relative abundance of AMR genes. **Table S4,** *Proteobacteria* populations strongly correlated with the *Proteobacteria* ratio. **Table S5.** Functional genes significantly correlated with *Proteobacteria* ratio (PLS). **Table S6.** Cluster distribution of functional genes significantly different between diets.
