# Supplementary material for: The rumen microbiome as a reservoir of antimicrobial resistance and pathogenicity genes is directly affected by diet in beef cattle
Source: Microbiome. 2017 Dec 11;5:159. doi: 10.1186/s40168-017-0378-z (PMC5725880; doi:10.1186/s40168-017-0378-z)
Supplement: Additional file 1: Figure S1. — Relative abundance (%) of 20 groups of functional genes representing 204 selected genes (number of animals, n = 50 samples). The sum of the relative abundance (%) of genes grouping within the same function is shown in this figure. Figure S2A. Total abundance of 204 selected genes based on diet treatments (n = 50). *P value < 0.05. Figure S2B. Shannon index diversity of 204 selected genes based on diet treatments (n = 50). *P value < 0.05, °P value < 0.1. Figure S3. Canonical Variate analysis (CVA) on the structure of 204 genes selected based on breed, age, weight, Proteobacteria ratio, FCR and methane grouping (n = 50). Figure S4. Factors explaining the significant differences observed for Proteobacteria ratio (n = 50). Figure S5. Microbial community composition at the phylum level (n = 50). Table S1. Characteristics of the cattle used in the experiment. Table S2. Groups of AMR genes significantly correlated with abundance of the Proteobacteria phylum and Proteobacteria ratio. Table S3. The relative abundance of AMR genes. Table S4, Proteobacteria populations strongly correlated with the Proteobacteria ratio. Table S5. Functional genes significantly correlated with Proteobacteria ratio (PLS). Table S6. Cluster distribution of functional genes significantly different between diets. (DOCX 60 kb) [file 40168_2017_378_MOESM1_ESM.docx]

**The rumen microbiome as a reservoir of antimicrobial resistance and pathogenicity genes is directly affected by diet in beef cattle**

Marc D. Auffret*^1^, Richard J. Dewhurst^1^, Carol-Anne Duthie^1^, John A. Rooke^1^, R. John Wallace^2^, Tom C. Freeman^3^, Robert Stewart^4^, Mick Watson^3,4^, Rainer Roehe^1^.

* Corresponding author

[Marc.Auffret@sruc.ac.uk](mailto:Marc.Auffret@sruc.ac.uk); [Richard.Dewhurst@sruc.ac.uk](mailto:Richard.Dewhurst@sruc.ac.uk); [Carol-Anne.Duthie@sruc.ac.uk](mailto:Carol-Anne.Duthie@sruc.ac.uk); [John.Rooke@sruc.ac.uk](mailto:John.Rooke@sruc.ac.uk); [john.wallace@abdn.ac.uk](mailto:john.wallace@abdn.ac.uk); [tfreeman@roslin.ed.ac.uk](mailto:tfreeman@roslin.ed.ac.uk); [Rob.Stewart@ed.ac.uk](mailto:Rob.Stewart@ed.ac.uk); [mick.watson@roslin.ed.ac.uk](mailto:mick.watson@roslin.ed.ac.uk); [Rainer.Roehe@sruc.ac.uk](mailto:Rainer.Roehe@sruc.ac.uk)

^1^ SRUC, Edinburgh, EH25 9RG, United Kingdom, ^2^ Rowett Institute, University of Aberdeen, Aberdeen, AB25 2ZD ,United Kingdom, ^3^ Division of Genetics and Genomics, The Roslin Institute and R(D)SVS, University of Edinburgh, Edinburgh, EH25 9RG ,United Kingdom, ^4^ Edinburgh Genomics, The Roslin Institute and R(D)SVS, University of Edinburgh, Edinburgh, EH25 9RG, United Kingdom.

**Abstract**

**Background: The emergence and spread of antimicrobial resistance is the most urgent current threat to human and animal health.** An improved understanding of the abundance of antimicrobial resistance genes and genes associated with microbial colonization and pathogenicity in the animal gut will have a major role in reducing the contribution of animal production to this problem. Here, the influence of diet on the ruminal resistome and abundance of pathogenicity genes was assessed in ruminal digesta samples taken from 50 antibiotic-free beef cattle, comprising four cattle breeds receiving two diets containing different proportions of concentrate.

**Results:** Two hundred and four genes associated with antimicrobial resistance (AMR), colonization, communication or pathogenicity functions were identified from 4966 metagenomic genes using KEGG identification. Both the diversity and abundance of these genes were higher in concentrate-fed animals. Chloramphenicol and microcin resistance genes were dominant in samples from forage-fed animals (*P*<0.001; Fig 2b), while aminoglycoside and streptomycin resistances were enriched in concentrate-fed animals. The concentrate-based diet also increased the relative abundance of *Proteobacteria*, which includes many animal and zoonotic pathogens. A high ratio of *Proteobacteria* to (*Firmicutes* + *Bacteroidetes*) was confirmed as a good indicator for rumen dysbiosis, with 8 cases all from concentrate-fed animals. Finally, network analysis demonstrated that the resistance/pathogenicity genes are potentially useful as biomarkers for health risk assessment of the ruminal microbiome.

**Conclusions:** Diet has important effects on the complement of AMR genes in the rumen microbial community, with potential implications for human and animal health.

**Keywords: Rumen microbiome, AMR, diets, Metagenomics, *Proteobacteria* ratio.**

**BACKGROUND**

Two significant challenges face mankind in the near future. The first is to feed a human population that may soon exceed nine billion people [1], the second is to deal with increasing medical and veterinary problems arising from the emergence of antimicrobial-resistant (AMR) pathogenic bacteria [2]. Ruminant livestock production is highly relevant to both issues. Ruminants consume feedstuffs, including forages and by-products, that cannot be used by non-ruminants. Thus, ruminant products provide high-quality nutrients from materials that do not compete with human-edible food. However, the ruminant digestive tract contains AMR genes that may be a reservoir from which AMR develops and spreads [2-4]. The use of antimicrobial feed additives increases the abundance of AMR genes in the gut and faeces [4, 5], particularly in beef cattle [3, 6]. Indeed, more antimicrobials are used in livestock production that in human therapeutics [7]. AMR genes in the ruminant gut are not entirely due to antimicrobial use, but appear also in non-medicated animals [8, 9]. Given the overarching importance of diet in determining the composition of the ruminal microbiome [10, 11], we considered that the absence of knowledge about the influence of diet on the ruminal ‘resistome’ (the collection of all identified antibiotic resistance genes) was a deficiency in understanding how AMR develops and spreads in livestock. A diet change (e.g. forage to concentrate) could generate a stress for the rumen microbiome community [12] as similarly found in humans by Brown et al. [13]. Thus, we analysed metagenomics data from beef cattle which had previously been used to explore links between the host animal, its microbiome/metagenome and methane emissions and feed efficiency.

Dietary changes could also potentially initiate a “bloom” of specific microbial populations, mostly Proteobacteria [14] or enhance the abundance of stress-response genes within the microbial community [15]. This unbalanced microbial community, also known as “dysbiosis”, is generally observed after diet changes and alterations in ruminal volatile fatty acid (VFA) composition also potentially associated with a lower ruminal pH, antibiotic treatment, presence of heavy metals or toxic substances or infection with pathogenic bacteria [13, 16]. For example, a low acetate-to-propionate (A:P<2.2) ratio is associated with a lower ruminal pH in animals offered a high-concentrate diet [17]. All of these factors can have negative effects on the overall health and production performance of cattle [18, 19]. *Proteobacteria* is one of the four main phyla in the rumen microbiome together with *Firmicutes*, *Bacteroidetes*, and *Actinobacteria*. This phylum is composed of many pathogenic bacteria such as *Escherichia coli* and the abundance of some of these opportunistic pathogens is sensitive to dietary change [20]. Diet has been shown to have a dominant effect on the shedding of the zoonotic pathogen, *E. coli* O157, from beef cattle [21]. Thus, the combined effects of pathogen abundance and the abundance and diversity of AMR genes have fundamental implications for human health.

Finally, microbial mechanisms including quorum sensing and fucose sensing [22, 23] are recognized as inter-kingdom signalling pathways [24], and help to regulate bacterial colonization and virulence within the host [25].

Our study design allowed us to test the impact of diet (“concentrate” versus “forage”) and host influence (e.g. breed, age, or weight) on the ruminal microbiota and functional genes associated with the microbiome [26]. Metagenomics was already confirmed as a good strategy to identify links between the rumen microbiome and host response [20, 27].

Therefore, the overall aim of our work was to improve knowledge on the diversity and importance of resistance- and pathogenicity-related genes as well as microbiome-host interaction genes in the rumen microbial community affected by diet treatments and over three independent trials. The factors influencing the composition of these genes in the rumen microbiome were evaluated and the genes showing significant differences were identified. We hypothesized that the microbiome of cattle offered a concentrate-based diet will favour stress associated with acidotic challenge and therefore enriched in microbial genes related to the resistome or pathogenicity activities, when compared to the cattle offered the forage-based diet. Rumen samples with higher Proteobacteria content, reaching a dysbiosis state, were of particular interest for understanding its association with diet treatments and how this can be explained by enrichment of the targeted genes. We also tried to identify a possible link between these genes and the control that the host has on its microbiome. This could be an important step towards identifying novel biomarkers of possible host-microbiome interactions involving beneficial and detrimental effects on the health of the host and related to diet change.

**MATERIALS AND METHODS**

**Animals, experimental design and diets**

In our previous study [26, 28], data on feed efficiency and methane emissions (measured using respiration chambers) were obtained from a 2 × 2 factorial design experiment of breed types and diets using 72 steers from a two-breed rotational cross between Aberdeen Angus (AA) and Limousin (LIM) and completed in 2011. Similar experiments were repeated using purebred Luing (LU) and crossbred Charolais (CH) steers in 2013 and Aberdeen Angus (AA) and Limousin (LIM) rotational crossbred steers in 2014. Prior to start of the experiment, all animals received the same diet type (Forage-based diet) and thereafter were adapted to the experimental diets over 5 weeks. During the same period, they were also acclimatized to the group-housed environment and the electronic feeders (HOKO, Insentec, Marknesse, The Netherlands). Due to EU legislation, the application of antibiotics is prohibited for enhancing growth. In exceptional cases, animals were treated with antibiotics and then excluded from the trial. Finally, there was not change in diets of the animals and was no fasting period before the animals went to slaughter.

The data in this study were samples from those experiments whereby animals with extreme high and low methane emissions (2011) or feed conversion efficiency (2013 and 2014) were selected for whole genome sequencing. The breed type of the sampled progenies were balanced within experiment comprising of 4 AA and LIM in 2011, 9 LU and CH in 2013 and 12 AA and LIM in 2014. The animals were offered two complete diets *ad libitum* containing (g/kg DM) approximately 500 forage to 500 concentrate or 80 forage to 920 concentrate subsequently referred to as forage and concentrate diets, respectively (Table S1). Full details of diets are given in Rooke et al. [11] and Duthie et al. [29, 30]. Animals from the 2014 experiment were only offered the forage diet (see Table S1) and this experiment was included in our analysis to increase observations for the forage diet, which is more heterogeneous in composition than the concentrate diet and to reinforce the microbiome results obtained with similar forage diets for the 2011 and 2013 experiments. A single sample of rumen fluid for VFA analysis (expressed as molar proportions) was taken by stomach tube within 1 h of cattle leaving the respiration chambers in the 2011 experiment [11]. VFA were determined for 2013 and 2014 in samples collected directly at the abattoir [11]. As recommended by Terré et al. [31], we compared VFA profiles between samples rather than total VFA concentrations because of the different methods for rumen sampling applied. The acetate-to-propionate ratio was calculated and considered as a proxy for rumen pH, accepting that whilst the relationship between the two is generally strong, it is not exactly linear. A total of 50 animals including the 8 animals already studied in Roehe et al. [26], selected and balanced for breed type and diet were used for further analyses (Table S1). Post-mortem digesta samples were used here, following our previous discovery that the abundance of archaea relative to bacteria was similar between live and slaughter cattle [32].

**Genomic analysis**

As previously described in Roehe et al. [26], the animals were slaughtered in a commercial abattoir where two rumen fluid samples (approximately 50 mL) were taken immediately after the rumen was opened to be drained. DNA was extracted from the rumen samples following the protocol described in Rooke et al. [11].

Illumina TruSeq libraries were prepared from genomic DNA and sequenced on Illumina HiSeq systems 2500 (2011) and 4000 (2013, 2014) by Edinburgh Genomics. Further analyses using the two sets of data followed the same procedure as previously described in Roehe et al. [26]. Briefly, microbial functional genes encoding for proteins and including the genes detailed in this study were identified using the KEGG genes database. Genes with a relative abundance of more than 0.001% were selected for further analysis.

A specific analysis was carried out on antimicrobial resistance genes and a comparison between the annotation obtained using KEGG genes and the genotyping tool SRST2 associated with the ARG-ANNOT database was performed; the latter was created specifically to study AMR genes [33, 34]. ARG-ANNOT targets all AMR genes including those on the chromosome and offers the possibility to identify putative new AMR genes in bacterial genomes as well as the genes involved in regulatory function associated with the AMR genes [35] and still showing high sensitivity and specificity for these genes, similar to other databases (e.g. ResFinder database) [36].

As for the analysis of metagenomics data using KEGG, a matrix was generated using the depth values and normalizing the relative abundance of each AMR genes per sample. The length and coverage values for AMR genes were on average 1386±26bp and 99.31±0.08% respectively.

For 16S rRNA gene analysis, the genomic reads were aligned to the GREENGENES database [37] using Novoalign (www.novocraft.com). The Proteobacteria ratio was calculated as the abundance of *Proteobacteria* sequences divided by the sum of the abundance of *Firmicutes* and *Bacteroidetes* sequences and is used as indicator for microbial disorder in both the rumen of cattle and human intestines [12, 14]. Values equivalent or above 0.19 were considered as an indication of the microbial disorder also known as dysbiosis. This cut-off value was calculated based on the abundance of the bacterial phyla identified in the core rumen microbiome of cattle fed forage or concentrate diets [12] and confirmed as a marker of microbial dysbiosis in the human gut [14].

Parameters were adjusted such that all hits were reported that were equal in quality to the best hit for each read, and allowing up to a 10 % mismatch across the fragment. Further details are included in Roehe et al. [26].

**Statistical analysis**

Statistical analysis of the metagenomic data was based on the complete sample profiles as expressed by the pattern of metagenomic operational functional units (OFUs) and the relative abundance (percentage) of individual OFU in each profile. Principal coordinate analysis (PCoA) and canonical variate analysis (CVA) were carried out using Gen-Stat 16th edition (VSN International Ltd, UK) to identify the factor(s) explaining the difference observed in microbial functional genes between samples. In CVA, each factor was tested individually in order to get a more constrained analysis.

Microbial functional gene abundances were compared individually or within a functional group (e.g. all genes associated with antibiotic resistance) using a general linear model with adjustment for multiple comparisons using the Bonferroni correction (SPSS Statistics 22, IBM, USA). Spearman’s correlations were carried out using SPSS Statistics 22 (IBM, USA) to identify the correlation between the AMR gene groups and *Proteobacteria* phylum or ratio, as well as between the *Proteobacteria* ratio and 20 *Proteobacteria* families or genera. *P*-values≤0.05 were considered significant and tendencies were reported (*P*-values<0.1).

In a network analysis using BioLayout Express3D [38], we identified the distinct functional clusters of microbial genes for each experiment and the combination of the three experiments. These networks consist of nodes representing microbial genes and the connecting edges determining the correlations in abundance and functional linkages between these genes.

We used partial least squares analysis (PLS, Version 9.1 for Windows, SAS Institute Inc., Cary, NC, USA) to identify the most important genes associated with the *Proteobacteria*:(*Firmicutes*+*Bacteroidetes*) ratio. The PLS analysis accounts for multiple testing and the correlation between microbial genes. In addition to microbial genes, the model included the effects of diet (potentially stressor) and breed type (host genetics effect). The model selections were based on the variable importance for projection (VIP) criterion [39], whereby microbial genes with a VIP<0.8 contribute little to the prediction.

**RESULTS**

**Major factors influencing functional genes in the microbiome**

Two hundred and four genes associated with resistance, colonization, communication or pathogenicity functions were identified from 4966 metagenomic genes using KEGG identification. These genes represent on average 1.07% of the total microbial genes in the rumen samples and could be classified into 20 functional groups (Fig S1). The total number of selected genes was significantly higher (*P*=0.046) in rumen samples from concentrate-fed compared to forage-fed cattle (Fig S2A) and diversity was also greater in concentrate-fed steers (Fig S2B). Biofilm formation and antibiotic resistance genes, representing 46.76% of the 204 genes, were the dominant groups detected. Other genes such as those encoding for quorum sensing only represented 0.1% of the 204 genes initially selected. Differences in gene composition observed between rumen samples and their clustering due to diets was confirmed by PCoA that explained 68.6% over the first two axes (Fig 1A). After grouping the samples, CVA confirmed that diet was the only factor that was significantly correlated (no overlapping within the 95% circles of confidence) with the functional gene composition and explaining this difference (Fig 1B). Host breed effect and *Proteobacteria* ratio were not significantly correlated with functional gene composition (Fig S3).

The abundance of 50 genes, each representing at least 0.001% of total genes per sample, were identified as different between diets (*P*<0.05; Fig 2). A greater number of genes (n=32) were more abundant in samples from animals offered the concentrate diet (n=18). Opposite results were observed between genes with similar function like AMR genes (Fig 3). For example, genes associated with resistance to macrolide (K08217) and β-lactamase (K01467, K07576) were significantly more abundant in samples from animals offered the concentrate diet (*P*<0.001), whilst the sum of genes including some specific genes related to chloramphenicol (K00638) and microcin resistance were dominant in forage-fed samples (*P*<0.001; Fig 3). Finally, genes encoding for aminoglycoside (K00897) and streptomycin (K00984) resistance were more abundant in concentrate treatment samples (*P*<0.01; Figs 2, 3). In general, similar results were confirmed when using the more specific AMR genes database. The only difference was the identification of genes encoding for tetracycline resistance instead of chloramphenicol resistance, which were in general significantly more abundant (*P*<0.01) in forage-fed animals (Table S2, S3).

The relative abundance of genes associated with stress responses including oxidative stress and cold shock protein (K03704) differed between diets. For example, K03704 was 3-fold more abundant with the concentrate diet. The concentrate diet enhanced the relative abundance of genes associated with motility, attachment to host cells, sensing (two-component signal transduction systems) and transporter for iron acquisition (K04758, K04759). The forage diet enhanced several genes - encoding for a type IV pilus assembly protein (K02652, K02662 to K02664 and K02666). Finally, type I and II secretion systems (K01993, K02456) were significantly more abundant in samples from forage-fed animals, whilst the gene associated with a type VI secretion system was enriched in concentrate-fed samples (K11907).

**Identification of markers of rumen microbial disorder and their link with diet effects on the microbiome**

Of the 24 most abundant archaeal and bacterial phyla, 12 phyla including *Proteobacteria* were more abundant in animals offered the concentrate diet (*P*<0.1; Figs 4 and S5). In contrast, *Euryarchaeota*, *Planctomycetes, Fibrobacteres* and *Verrucomicrobia* were more abundant in rumen samples from animals offered a forage diet (*P*<0.01). *Firmicutes*, *Bacteroidetes* and 14 other phyla were not significantly affected by diet. A *Proteobacteria* ratio above 0.19, suggesting dysbiosis, was only detected in rumen samples from the concentrate diet treatment and thus this ratio was on average higher for rumen samples from animals offered the concentrate than the forage diet (*P*=0.001; Fig 5). In addition, this ratio was strongly correlated with diet change (*P*=0.001), but not with other factors tested except methane emissions (Fig S4). In addition, a significant negative correlation was found between the Acetate:Propionate ratio, a proxy for low ruminal pH (lower ratio) and the *Proteobacteria* ratio when analysing all samples (Fig 6). In general, A:P ratio from rumen samples associated with forage grouped together with an average value of 3.77±0.66 (Fig 6c).

A high proportion of populations explaining this higher *Proteobacteria* ratio were composed of families (7/20) and genera (7/20) known to be pathogens in animals, humans, or even in plants (Table S4) and were strongly correlated with the *Proteobacteria* ratio (*P*<0.001). For example, *Aeromonadaceae* including the *Aeromonas* genus and *Enterobacteriaceae* were the most abundant Proteobacteria populations known to contain pathogens. Other populations were identified as gut commensals, or with antimicrobial activities, or linked with nutrients (N, S or Fe) or organic matter degradation capacities.

Over the three experiments, 43 genes identified by PLS analysis were considered important in predicting the impact of the *Proteobacteria* ratio on the microbial community (Table S5). The relative abundance of the selected genes explained 48% of the factor fitted in the model and 81% of the *Proteobacteria* ratio. Of these genes, 22 genes were also affected by the dietary treatment – including genes encoding for beta-lactamase (K01467, K07576) and cold shock protein (K03704). For each experiment, a network was generated based on the total KEGG identified genes (n=4966) including on average 56 genes from the 204 genes and representing about 4.36% of all genes constituting the network. Although these genes were distributed over several clusters, about half of the genes (54%) grouped into one or two dominant clusters when studied per experiment (Table S5-S6) and still grouped in two dominant clusters when the network analysis combined all experiments (Fig 7). The most abundant genes (relative abundance above 0.01%) within these two clusters encoded for flagellin (K02406), beta-lactamase (K07576) and Type VI secretion system (K11907).

**DISCUSSION**

In the present study, the results of three independent experiments were combined. This meta-analysis was used to highlight the importance of diet rather than the host on the structure of rumen microbial genes associated with resistance, colonization, communication and pathogenicity activities. It is known that diet has a dominant effect on the microbial community composition [40 41] and here we first identified that diet influences functional genes associated with AMR and more interestingly microbial genes (e.g. genes encoding for cold shock protein, T6SS, motility, hooking and sensing) allowing the microbial populations to adapt to the new ruminal environment generated by concentrate diet. Information on the composition of the initial rumen microbiome may provide further information, but was not available for this study. However, all animals were offered the same forage-based diet prior to the trial and animals were randomly allocated to diets (balanced for breed) at the start of each experiment. Consequently, there is no reason to believe that the prevalence of antibiotic resistance genes differed at the start of each experiment. Therefore, we are confident in our results that concentrate diet significantly increased the relative abundance and diversity of AMR genes and other genes studied in this manuscript.

The greater abundance of genes encoding for sensing (two-component signal transduction systems) or secretion system (T6SS) in concentrate diet samples confers advantages for microorganisms to sense, respond, and adapt to a wide range of environments [42] using different mechanisms [43-45]. T6SS which is more prevalent in *Proteobacteria* helps in colonizing the gut [46] and controlling bacterial growth in a disturbed rumen [47]. Moreover, microorganisms carrying genes encoding flagellin, which are enhanced when a high-concentrate diet is fed, are known to develop strategies to evade innate immune system detection [48, 49] and therefore increase the risk of disease. Another host-microbiome interaction system involves fucose sensing and one gene encoding for alpha-L-fucosidase was significantly enriched in concentrate-fed animals. This gene may enhance the abundance of commensals and also pathogenic bacteria carrying this gene and increase the risk of infection [22]. Finally, it is known that competition between host and pathogens occurs for iron resources. Genes associated with iron storage (ferritin) or transport were differently affected depending of the diet and tend to suggest a lower iron availability for microorganisms including pathogens in concentrate-compared to forage-fed animals. It is known that iron serves as a signal for bacterial pathogens to recognise host cells in an iron-depleted environment, potentially leading to disease [50].

Henderson et al. [10] found similar results, demonstrating the predominant influence of diet over the host effect and explaining the differences in microbial community composition. The host effect was identified in the present study as a breed effect, which has been shown in our previous study to be of substantially less impact on the microbiome, than within-breed genetic effects.

In addition, feeding the concentrate diet significantly enhanced the diversity of these rumen microbial genes (higher evenness) including a broader spectrum of mechanisms of resistance compared to the forage diet. The increase in abundance and diversity of AMR and pathogenicity genes in rumen samples from concentrate-fed animals could also be the result of multi-resistant bacteria enrichment [51], or an increase in horizontal gene transfer (HGT) as a result of gut dysbiosis [52, 53] or rumen colonization [54]. Therefore, the risk of spreading antimicrobial resistance genes between microbial populations belonging to the same or a different phylum and including human pathogens increases [55].

Microbial genes significantly abundant in rumen samples from animal offered the forage diet were mainly associated with activities known to be involved in beneficial host-microbiome interaction and its homeostasis involving non-pathogenic bacteria. For example, antimicrobial peptides like microcin are produced and released by gut epithelial cells and help to maintain the host-microbiome homeostasis [56]. Homeostasis helps to reduce pathogen colonization [57] without causing collateral damage to host cells [49; 53]. Therefore, it is coherent to find more microcin-resistance genes mostly identified in commensal bacteria [58] and also carrying microcin synthesis genes providing a competitive advantage against enteric pathogens [57] when feeding a high-forage diet, which is known to promote rumen homeostasis [59]. This system is less aggressive and energy demanding than other systems like T6SS used by e.g. pathogenic *Proteobacteria* [45]. Forage diets, which contain more fibre, are recognised to enhance gut health in different animal species by modulating the gut microbiome, its impact on the host mucosa and reducing the level of stress or disease compared to grain-fed animals [59; 60].

The results obtained with the concentrate diet can be explained by the necessity for the rumen microbiome to adapt to a new environment [61] and the increase in populations with broader adaptability and colonization capacities. Interestingly, the relative abundance of *Proteobacteria*, one phylum containing bacterial populations associated with AMR and pathogenicity [27] was one of the bacterial phyla enriched when feeding the concentrate diet. We also identified bacterial genera within *Proteobacteria* (e.g. *Tolumonas* or *Methylomonas)* and including pathogens (e.g. *Aeromonas* or *Moraxella)* with a relative abundance above 0.001% and strongly correlated with the *Proteobacteria* ratio which may explain the high *Proteobacteria* ratio detected in 8 rumen samples from animal offered the concentrate diet. Interestingly, the plant pathogen *Pantoea*, belonging to *Proteobacteria* was also significantly more abundant when feeding the concentrate diet and highly correlated with the *Proteobacteria* ratio, suggesting that microorganisms colonizing plants and present in the diet might play a role in the rumen. Functional similarities between microorganisms inhabiting the gut or plant roots and sharing similar ecological niches were found, supporting a potential role of these plant-associated microbial populations during the acidotic challenge [62].

*Proteobacteria* populations partly explained the change in functional gene composition and diversity observed in samples from animals offered the concentrate diet. A high *Proteobacteria* ratio was also significantly negatively correlated with a low acetate:propionate ratio (a proxy for low ruminal pH) [12]. It is known that concentrate diets with a high grain content tend to increase propionate concentration and are associated with lower rumen pH, as well as an increase in the abundance of *Proteobacteria* [12]. The formulation of concentrate diets with respect to the proportion of starch to digestible fibre could be more important than the concentrate type for explaining the effect on rumen pH [63] and this information could be used as a strategy to reduce the detrimental effect of concentrate diet increasing the abundance and diversity of the genes targeted in this study.

We confirmed the relevance of using a *Proteobacteria* ratio instead of *Proteobacteria* abundance as a proxy of a microbiome dysbiosis and more pathogenic bacteria may increase the risk of host disease [12, 14]. This ratio was significantly correlated with a gene encoding for a cold shock protein known to have a wider role in bacterial stress tolerance and reinforcing the detrimental effect of the concentrate diet on the rumen microbiome [2].

Finally, the 43 rumen microbial genes identified by PLS analysis to be significantly associated with the *Proteobacteria* ratio, include several AMR genes such as genes encoding for beta-lactamase. Although, these genes were distributed over several clusters, most of them grouped in one major cluster as shown by the network analysis. Therefore, it would be possible to apply the same strategy as Roehe et al. [26] to determine a baseline of these genes for risk assessment.

**CONCLUSIONS**

In conclusion, diets with high fibrous forage components would be recommended over those with high grain components to lower the abundance and diversity of AMR genes and to avoid rumen microbiome dysbiosis associated with pathogenic species among ruminal *Proteobacteria*. The results presented here will help to improve policies about antimicrobial treatments in beef cattle production and to help avoid AMR gene transmission to bovine-associated human pathogens. We describe AMR gene abundance/diversity in antibiotic-free cattle that complements published information on AMR genes in animals receiving antimicrobials. It will now be important to combine the investigation of AMR usage with dietary regime, to determine whether alterations in diet can lessen the threats associated with AMR in livestock production.

**DECLARATIONS**

### Ethics approval and consent to participate

This study was conducted at the Beef and Sheep Research Centre of Scotland’s Rural College (6 miles south of Edinburgh, UK). The experiment was approved by the Animal Experiment

Committee of SRUC and was conducted in accordance with the requirements of the UK Animals (Scientific Procedures) Act 1986.

### Consent for publication

### 'Not applicable'

### Availability of data and materials

These data can be downloaded from the European Nucleotide Archive under accession PRJEB10338 and PRJEB21624.

### Competing interests

"The authors declare that they have no competing interests".

### Funding

The project was supported by grants from the Biotechnology and Biological Sciences Research Council (BBSRC BB/N01720X/1 and BB/N016742/1). The research is based on data from experiments funded by the Scottish Government as part of the 2011-2016 commission, Agriculture and Horticulture Development Board (AHDB) Beef & Lamb, Quality Meat Scotland (QMS) and Department for Environment Food & Rural Affairs (Defra).

### Authors' contributions

Conceptualization: MDA and RR

Formal analysis: MDA and MW

Original writing: MDA and RR

Review and Editing: MDA, RJD, C-AD, JAR, RJW, TCF, RS, MW and RR

“All authors read and approved the final manuscript.”

**Acknowledgements**

We thank Dr Irene Cabeza Luna, Andrew Southwell, Asier Zaragoza, Laura Nicoll, Lesley Deans, and Claire Broadbent for the excellent technical support.

**REFERENCES**

1. Godfray HCJ, Beddington JR, Crute IR, Haddad L, Lawrence D, Muir JF. Food security: The challenge of feeding 9 billion people. Science 2010, 327:812-818.

2. Cameron A, McAllister TA. Antimicrobial usage and resistance in beef production. Journal of Animal Science and Biotechnology. 2016;7:68.

3. Noyes NR, Yang X, Linke LM, Magnuson RJ, Cook SR, Zaheer R, Yang H, Woerner DL, Geornaras I, McArt JA et al. Characterization of the resistome in manure, soil and wastewater from dairy and beef production systems. Sci Rep. 2016;6:24645.

4. Penders J, Stobberingh EE, Savelkoul PH, Mand Wolffs PFG. The human microbiome as a reservoir of antimicrobial resistance. Front Microbiol. 2013;4:87.

5. Reddy B, Singh KM, Patel AK, Antony A, Panchasara HJ, Joshi CG. Insights into resistome and stress responses genes in *Bubalus bubalis* rumen through metagenomic analysis. Mol Biol Rep. 2014;41:6405–6417.

6. Cameron A, McAllister TA. 2016. Antimicrobial usage and resistance in beef ` production. J Anim Sci Biotechnol, 2016;7:68.

7. Spellberg B, Hansen, GR, Kar, A, Cordova, CD, Price, LB, Johnson JR. 2016. Antibiotic resistance in humans and animals. Discussion Paper, National Academy of Medicine, Washington, DC. http://www.nam.edu/antibiotic-resistance-in-humans-and-animals.

8. Poole K. Bacterial stress responses as determinants of antimicrobial resistance. J Antimicrob Chemother. 2012;67:2069-2089.

9. Chambers L, Yang Y, Littier H, Ray P, Zhang T, Pruden A. Metagenomic analysis of antibiotic resistance genes in dairy cow feces following therapeutic administration of third generation cephalosporin. PLoS ONE. 2015;10:e0133764.

10. Henderson G, Cox F, Ganesh S, Jonker A, Young W, Collaborators. GRC, Janssen PH. Rumen microbial community composition varies with diet and host, but a core microbiome is found across a wide geographical range. Sci Rep. 2015;5:14567.

11. Rooke JA, Wallace RJ, Duthie C-A, McKain N, Motta de Souza S, Hyslop JJ, Ross DW, Waterhouse T, Roehe R. Hydrogen and methane emissions from beef cattle and their rumen microbial community vary with diet, time after feeding and genotype. British Journal of Nutrition. 2014;112:398-407.

12. Petri RM, Schwaiger T, Penner GB, Beauchemin KA, Forster RJ. Characterization of the core rumen microbiome in cattle during transition from forage to concentrate as well as during and after an acidotic challenge. PLoS ONE. 2013;8:e83424.

13. Brown K, DeCoffe D, Molcan E, Gibson DL. Diet-induced dysbiosis of the intestinal microbiota and the effects on immunity and disease. Nutrients 2012, 4:1095-1119.

14. Shin NR, Whon TW, Bae JW. Proteobacteria: microbial signature of dysbiosis in gut microbiota. Trends Biotechnol. 2015;33:496-503.

15. Keto-Timonen R, Hietala N, Palonen E, Hakakorpi A, Lindström M, Korkeala H. Cold Shock Proteins: A minireview with special emphasis on Csp-family of enteropathogenic *Yersinia*. Front Microbiol. 2016;7:1151.

16. Hawrelak J, Myers S. The causes of intestinal dysbiosis: a review. Altern Med Rev. 2004;9:180-197.

17. Russell JB. The importance of pH in the regulation of ruminal acetate to propionate ratio and methane production in vitro. [J Dairy Sci.](https://www.ncbi.nlm.nih.gov/pubmed/9891267) 1998;81:3222-30.

18. Koboziev I, Reinoso Webb C, Furr KL, Grisham MB. Role of the enteric microbiota in intestinal homeostasis and inflammation. Free Radic Biol Med. 2014;68:122-133.

19. Pitta DW, Parmar N, Patel AK, Indugu N, Kumar S, Prajapathi KB. Bacterial diversity dynamics associated with different diets and different primer pairs in the rumen of Kankrej cattle. PLoS ONE. 2014;9:e111710.

20. Bäumler AJ, Sperandio V. Interactions between the microbiota and pathogenic bacteria in the gut. Nature. 2016;535:85-93.

21. Callaway TR, Carr MA, Edrington TS, Anderson RC, Nisbet DJ. Diet, Escherichia coli O157:H7, and cattle: a review after 10 years. Curr Issues Mol Biol. 2009;11:67-79.

22. Pickard JM, Maurice CF, Kinnebrew MA, Abt MC, Schenten D, Golovkina TV, Bogatyrev SR, Ismagilov RF, Pamer EG, Turnbaugh PJ, Chervonsky AV. Rapid fucosylation of intestinal epithelium sustains host-commensal symbiosis in sickness. Nature. 2014;514:638-641.

23. Hughes DT, Terekhova DA, Liou L, Hovde CJ, Sahl JW, Patankar AV, Gonzalez JE, Edrington TS, Rasko DA, Sperandio V. Chemical sensing in mammalian host-bacterial commensal associations. Proc Natl Acad Sci U S A. 2010;107:9831-9836.

24. Curtis MM, Sperandio V. A complex relationship: the interaction among symbiotic microbes, invading pathogens, and their mammalian host. Mucosal Immunol. 2011;4:133-138.

25. Romero D, Traxler MF, López D, Kolter R. Antibiotics as signal molecules. Chem Rev. 2011;111:5492–5505.

26. Roehe R, Dewhurst RJ, Duthie C-A, Rooke JA, McKain N, Ross DW, Hyslop JJ, Waterhouse A, Freeman TC, Watson M et al. Bovine host genetic variation influences rumen microbial methane production with best selection criterion for low methane emitting and efficiently feed converting hosts based on metagenomic gene abundance. PLoS Genet. 2016;12:e1005846.

27. Durso LM, Miller DN, Wienhold BJ. Distribution and quantification of antibiotic resistant genes and bacteria across agricultural and non-agricultural metagenomes. PLoS ONE. 2012;7: e48325.

28. Wallace RJ, Rooke JA, McKain N, Duthie C-A, Hyslop JJ, Ross DW, Waterhouse A, Watson M, Roehe R. The rumen microbial metagenome associated with high methane production in cattle. BMC Genomics. 2015;16:839.

29. Duthie C-A, Rooke JA, Troy S, Hyslop JJ, Ross DW, Waterhouse A, Roehe R. Impact of adding nitrate or increasing the lipid content of two contrasting diets on blood methaemoglobin and performance of two breeds of finishing beef steers. Animal. 2016;10:786-795.

30. Duthie C-A, Troy SM, Hyslop JJ, Ross DW, Roehe R, Rooke JA. Feeding nitrate and increasing dietary lipid act independently as mitigation strategies to reduce methane emissions in finishing beef cattle offered forage based diets. Animal. accepted 21/05/17.

31. Terré M, Castells L, Fàbregas F. Short communication: Comparison of pH, volatile fatty acids, and microbiome of rumen samples from preweaned calves obtained via cannula or stomach tube. J Dairy Sci. 2013;96:5290–5294.

32. Wallace RJ, Rooke JA, Duthie C-A, Hyslop JJ, Ross DW, McKain N, Motta de Souza S, Snelling TJ, Waterhouse A, Roehe R. Archaeal abundance in postmortem ruminal digesta may help predict methane emissions from beef cattle. Sci Rep. 2014;4:5892.

33. Inouye M, Dashnow H, Raven LA, Schultz MB, Pope BJ, Tomita T, Zobel J, Holt KE. SRST2: Rapid genomic surveillance for public health and hospital microbiology labs. Genome Med. 2014;6:90.

34. Gupta SK, Padmanabhan BR, Diene SM, Lopez-Rojas R, Kempf M, Landraud L, Rolain J-M. ARG-ANNOT, a new bioinformatic tool to discover antibiotic resistance genes in bacterial genomes. Antimicrob Agents Chemother. 2014;58:212–220.

35. Gupta SK, Rolain J-M. Reply to "Comparison of the web tools ARG-ANNOT and ResFinder for detection of resistance genes in bacteria". Antimicrob Agents Chemother. 2014;58:4987.

36. Xavier BB, Das AJ, Cochrane G, De Ganck S, Kumar-Singh S, Aarestrup FM, Goossens H, Malhotra-Kumar S. Consolidating and exploring antibiotic resistance gene data resources. J Clin Microbiol. 2016;54:851– 859.

37. DeSantis TZ, Hugenholtz P, Larsen N, Rojas M, Brodie EL, Keller K. Greengenes, a chimera-checked 16S rRNA gene database and workbench compatible with ARB. Appl Environ Microbiol. 2006;72:5069–5072.

38. Freeman TC, Goldovsky L, Brosch M, van Dongen S, Mazière P, Grocock RJ. Construction, visualisation, and clustering of transcription networks from microarray expression data. PLoS Comput Biol. 2007;3: e206.

39. Wold S. PLS for multivariate linear modelling. Van de Waterbeemd H, editor Chemometric methods in molecular design VCH Publishers, Weinheim, Germany. 1995:195–218.

40. Chen Y, Penner GB, Li M, Oba M, Luo Guan L. Changes in bacterial diversity associated with epithelial tissue in the beef cow rumen during the transition to a high-grain diet. Appl Environ Microbiol. 2011;77:5770–5781.

41. Zhou M, Hernandez-Sanabria E, Guan LL. Characterization of variation in rumen methanogenic communities under different dietary and host feed efficiency conditions, as determined by PCR-denaturing gradient gel electrophoresis analysis. Appl Environ Microbiol. 2010;76:3776–3786.

42. Capra EJ, Laub MT. The evolution of two-component signal transduction systems. Annu Rev Microbiol. 2012;66:325–347.

43. Green ER, Mecsas J. Bacterial secretion systems: An overview. Microbiol Spectr. 2016;4:1.

44. Ho BT, Dong TG, Mekalanos JJ. A view to a kill: the bacterial type VI secretion system. Cell Host Microbe. 2014;15:9-21.

45. Ohland CL, Jobin C. Microbial activities and intestinal homeostasis: A delicate balance between health and disease. Cell Mol Gastroenterol Hepatol. 2015;1:28-40.

46. Barret M, Egan F, O'Gara F. Distribution and diversity of bacterial secretion systems across metagenomic datasets. Environ Microbiol Rep. 2013;5:117-126.

47. Asanuma N, Yoshii T, Kanada K, Yoshizawa K, Arai Y, Ichikawa T, Kawamura A, Hino T. Involvement of two-component signal transduction system, ComDE, in the regulation of growth and genetic transformation, in the ruminal bacterium *Streptococcus bovis*. Anaerobe. 2010;16:405-411.

48. Hayashi F, Smith KD, Ozinsky A, Hawn TR, Yi EC, Goodlett DR, Eng JK, Akira S, Underhill DM, Aderem A. The innate immune response to bacterial flagellin is mediated by Toll-like receptor 5. Nature. 2001;410:1099-1103.

49. Lai MA, Quarles EK, López-Yglesias AH, Zhao X, Hajjar AM. Innate immune detection of flagellin positively and negatively regulates *Salmonella* infection. PLoS ONE. 2013;8: e72047.

50. Skaar EP. The battle for iron between bacterial pathogens and their vertebrate hosts. PLoS PATHOGENS. 2010;6: e1000949.

51. Schjørring S, Krogfelt KA. Assessment of bacterial antibiotic resistance transfer in the gut. Int J Microbiol. 2011;2011:312956.

52. Aminov RI, Mackie RI. Evolution and ecology of antibiotic resistance genes. FEMS Microbiol Lett. 2007;271:147-161.

53. Stecher B, Maier L, Hardt WD. 'Blooming' in the gut: how dysbiosis might contribute to pathogen evolution. Nat Rev Microbiol. 2013;11:277-284.

54. Toomey N, Monaghan A, Fanning S, Bolton D. Transfer of antibiotic resistance marker genes between lactic acid bacteria in model rumen and plant environments. Appl Environ Microbiol. 2009;75:3146-3152.

55. Bliven KA, Maurelli AT. Evolution of bacterial pathogens within the human host. [Microbiol Spectr. 2016 Feb; 4(1): 10.](https://www.ncbi.nlm.nih.gov/entrez/eutils/elink.fcgi?dbfrom=pubmed&retmode=ref&cmd=prlinks&id=26999399)

56. Ostaff MJ, Stange EF, Wehkamp J. Antimicrobial peptides and gut microbiota in homeostasis and pathology. EMBO Mol Med. 2013;5:1465-1483.

57. Sassone-Corsi M, Nuccio SP, Liu H, Hernandez D, Vu CT, Takahashi AA, Edwards RA, Raffatellu M. Microcins mediate competition among *Enterobacteriaceae* in the inflamed gut. Nature. 2016;540:280-283.

58. Hejnova J, Dobrindt U, Nemcova R, Rusniok C, Bomba A, Frangeul L, Hacker J, Glaser P, SeboP, Buchrieser C. Characterization of the flexible genome complement of the commensal Escherichia coli strain A0 34/86 (O83 : K24 : H31). Microbiology. 2005;151:385-398.

59. Carrillo J, He Y, Li Y, Liu J, Erdman RA, Sonstegard TS, Song J. Integrated metabolomic and transcriptome analyses reveal finishing forage affects metabolic pathways related to beef quality and animal welfare. Sci Rep. 2016;6:25948.

60. Jha R, Berrocoso JD. Review: Dietary fiber utilization and its effects on physiological functions and gut health of swine. Animal. 2015;9:1441-1452.

61. Francino MP. The ecology of bacterial genes and the survival of the new. International Journal of Evolutionary Biology. 2012;2012:394026.

63. Ramírez-Puebla ST, Servín-Garcidueñas LE, Jiménez-Marín B, Bolaños LM, Rosenblueth M, Martínez J, Rogel MA, Ormeño-Orrillo E, Martínez-Romero E. Gut and root microbiota commonalities. Appl Environ Microbiol. 2013;79: 2–9.

63. Friggens NC, Oldham JD, Dewhurst RJ, Horgan G. Proportions of volatile fatty acids in relation to the chemical composition of feeds based on grass silage. J Dairy Sci. 1998;81:1331-1344.

**Figure captions**

Fig 1A: Principal Coordinates analysis (PCoA) of the structure of 204 selected genes (number of animals, n=50 samples).

Black cross: Concentrate samples from 2011 experiment, White cross: Concentrate samples from 2013 experiment, Black triangle: Forage samples from 2011 experiment, Dark grey triangle: Forage samples from 2013 experiment, Grey triangle: Forage samples from 2014 experiment.

Fig 1B: Canonical Variate analysis (CVA) of the structure of 204 selected genes (n=50) based on diet.

Black cross: Samples from concentrate-fed animals (all years), Grey triangle: Samples from forage-fed animals (all years). Circle: 95% confidence range.

Fig 2: Relative abundance of genes significantly different between diet treatments (n=50).

Mean values with standard error are presented.

Grey: Samples from forage-fed animals, Dark grey: Samples from concentrate-fed animals.

Arrow indicates the genes that are also detected in the network analysis.

Fig 3: Diversity of AMR genes between diets (n=50).

AMR genes with similar antibiotic resistance are grouped together into a final number of 8 groups.

Fig 4: Relative abundance of microbial phyla between diet treatments (n=50).

Grey: Samples from forage-fed animals, Dark grey: Samples from concentrate-fed animals.

***P-*value<0.01, **P-*value<0.05, °*P-*value<0.1.

Fig 5: Calculated *Proteobacteria* ratio over the three experiments (n=50).

Cutoff: Values above 0.19 are considered as an indicator of rumen dysbiosis.

Grey: Samples from forage-fed animals, Dark grey: Samples from concentrate-fed animals.

Fig 6: Linear regression for studying the impact of Acetate:Propionate ratio to *Proteobacteria* ratio. (a) All samples (n=50). (b) Samples from concentrate-fed animals (n=16). (c) Samples from forage-fed animals (n=34). Equation for the linear regression was included in figure when the difference was significant (*P*-value<0.05).

Fig 7: Functional clusters of AMR genes identified using network analysis combining the three independent experiments. Correlation analysis of microbial gene abundance was used to construct networks, where nodes represent microbial genes and edges the correlation in their abundance.
