# Supplementary material for: Correction to: The rumen microbiome as a reservoir of antimicrobial resistance and pathogenicity genes is directly affected by diet in beef cattle
Source: Microbiome. 2019 Nov 18;7:149. doi: 10.1186/s40168-019-0764-9 (PMC6862725; doi:10.1186/s40168-019-0764-9)
Supplement: Supplementary file 1 — Additional file 1: Figure S1.Relative abundance (%) of 20 groups of functional genes representing 204 selected genes (number of animals, n = 50 samples). The sum of the relative abundance (%) of genes grouping within the same function is shown in this figure. Figure S2A. Total abundance of 204 selected genes based on diet treatments (n = 50). *P value < 0.05. Figure S2B. Shannon index diversity of 204 selected genes based on diet treatments (n = 50). *P value < 0.05, °P value < 0.1. Figure S3. Canonical Variate analysis (CVA) on the structure of 204 genes selected based on breed, age, weight, Proteobacteria ratio, FCR and methane grouping (n = 50). Figure S4. Factors explaining the significant differences observed for Proteobacteria ratio (n = 50). Figure S5. Microbial community composition at the phylum level (n = 50). Table S1. Characteristics of the cattle used in the experiment. Table S2. Groups of AMR genes significantly correlated with abundance of the Proteobacteria phylum and Proteobacteria ratio. Table S3. The relative abundance of AMR genes. Table S4, Proteobacteria populations strongly correlated with the Proteobacteria ratio. Table S5. Functional genes significantly correlated with Proteobacteria ratio (PLS). Table S6. Cluster distribution of functional genes significantly different between diets. [file 40168_2019_764_MOESM1_ESM.zip › Auffret paper AMR Sup Material_Revised.docx]

**The rumen microbiome as a reservoir of antimicrobial resistance and pathogenicity genes is directly affected by diet in beef cattle**

Marc D. Auffret*^1^, Richard J. Dewhurst^1^, Carol-Anne Duthie^1^, John A. Rooke^1^, R. John Wallace^2^, Tom C. Freeman^3^, Robert Stewart^4^, Mick Watson^3,4^, Rainer Roehe^1^.

* Corresponding author

^1^ SRUC, Edinburgh, EH25 9RG, United Kingdom, ^2^ Rowett Institute, University of Aberdeen, Aberdeen, AB25 2ZD ,United Kingdom, ^3^ Division of Genetics and Genomics, The Roslin Institute and R(D)SVS, University of Edinburgh, Edinburgh, EH25 9RG ,United Kingdom, ^4^ Edinburgh Genomics, The Roslin Institute and R(D)SVS, University of Edinburgh, Edinburgh, EH25 9RG, United Kingdom.

**Supporting information**

Fig S1: Relative abundance (%) of 20 groups of functional genes representing 204 selected genes (number of animals, n=50 samples). The sum of the relative abundance (%) of genes grouping within the same function is shown in this figure.

Fig S2A: Total abundance of 204 selected genes based on diet treatments (n=50).

**P-*value<0.05

Fig S2B: Shannon index diversity of 204 selected genes based on diet treatments (n=50).

**P-*value<0.05, °*P-*value<0.1.

Fig S3: Canonical Variate analysis (CVA) on the structure of 204 genes selected based on breed, age, weight, *Proteobacteria* ratio, FCR and methane grouping (n=50).

Black cross: Samples from concentrate-fed animals from the three years, Grey triangle: Samples from forage-fed animals from the three years. Circle: 95% confidence range.

Fig S4: Factors explaining the significant differences observed for *Proteobacteria* ratio (n=50).

Conc: Concentrate, For: Forage, FCR: Feed conversion ratio, AA: Aberdeen Angus, CH: Charolais, LIM: Limousin, ***P-*value<0.01.

Fig S5: Microbial community composition at the phylum level (n=50).

Others: *Armatimonadetes, Caldiserica, Calditrichaeota, Candidatus Cloacimonetes, Chlamydiae, Chrysiogenetes, Crenarchaeota, Ignavibacteriae, Thaumarchaeota* and *Thermodesulfobacteria*.

Table S1. Characteristics of the cattle used in the experiment.

| Year Trial-Sample | Breed type | Diet | Age at entry to chamber (days) | Live-weight (kg) | FCR (kg intake/kg gain) | *Proteobacteria* ratio^1^ | Methane (g/kg DMI) | Acetate at slaughter mmol/mol | Propionate at slaughter mmol/mol | Acetate:  Propionate ratio  Mol/mol |
| --- | --- | --- | --- | --- | --- | --- | --- | --- | --- | --- |
| 2011 Experiment (Concentrate/Forage diet) | | | | | | | | | | |
| 2011-N1 | AA sired | CONC | 527 | 725 | 6.10 | 0.15 | 7.63 | N.D. | N.D. | N.D. |
| 2011-N2 | AA sired | CONC | 546 | 687 | 6.10 | 0.04 | 18.14 | 599 | 194 | 3.09 |
| 2011-N3 | Lim. sired | CONC | 498 | 693 | 9.33 | **0.23** | 9.29 | N.D. | N.D. | N.D. |
| 2011-N4 | Lim. sired | CONC | 546 | 654 | 8.04 | 0.05 | 20.13 | 567 | 243 | 2.33 |
| 2011-N5 | AA sired | FOR | 523 | 669 | 10.38 | 0.17 | 17.41 | 665 | 190 | 3.50 |
| 2011-N6 | AA sired | FOR | 526 | 681 | 6.72 | 0.02 | 32.42 | 725 | 113 | 6.42 |
| 2011-N7 | Lim. sired | FOR | 530 | 654 | 8.07 | 0.03 | 19.37 | N.D. | N.D. | N.D. |
| 2011-N8 | Lim. sired | FOR | 547 | 659 | 8.12 | 0.01 | 30.37 | 646 | 170 | 3.80 |
| 2013 Experiment (Concentrate/Forage diet) | | | | | | | | | | |
| 2013-RR0001 | LU sired | FOR | 483 | 674 | 8.67 | 0.02 | 17.96 | 660.1 | 168.6 | 3.91 |
| 2013- RR0002 | LU sired | FOR | 467 | 742 | 7.11 | 0.14 | 18.16 | 658.6 | 207.1 | 3.18 |
| 2013- RR0003 | CH sired | FOR | 435 | 714 | 8.70 | 0.04 | 25.97 | 664.0 | 158.8 | 4.18 |
| 2013- RR0004 | CH sired | FOR | 401 | 694 | 6.00 | **0.40** | 22.93 | 645.1 | 200.0 | 3.23 |
| 2013- RR0005 | LU sired | CONC | 482 | 600 | 8.57 | **0.22** | 18.85 | 530.9 | 313.3 | 1.69 |
| 2013- RR0006 | CH sired | CONC | 420 | 636 | 9.12 | **0.47** | 20.93 | 605.7 | 189.5 | 3.20 |
| 2013- RR0007 | CH sired | CONC | 472 | 670 | 5.85 | 0.16 | 12.70 | 590.4 | 264.2 | 2.23 |
| 2013- RR0008 | LU sired | CONC | 481 | 644 | 6.97 | 0.07 | 16.61 | 554.4 | 272.0 | 2.04 |
| 2013- RR0009 | LU sired | FOR | 493 | 671 | 9.65 | 0.04 | 29.64 | 684.2 | 193.8 | 3.53 |
| 2013- RR0010 | CH sired | FOR | 486 | 778 | 9.22 | 0.01 | 27.12 | 668.0 | 184.0 | 3.63 |
| 2013- RR0011 | CH sired | CONC | 417 | 702 | 7.00 | **0.19** | 15.28 | 561.5 | 213.1 | 2.64 |
| 2013- RR0012 | CH sired | CONC | 482 | 696 | 8.57 | 0.02 | 16.01 | 401.2 | 452.5 | 0.89 |
| 2013- RR0013 | LU sired | CONC | 490 | 696 | 11.54 | **0.21** | 18.16 | 567.4 | 292.1 | 1.94 |
| 2013- RR0014 | LU sired | CONC | 493 | 632 | 8.03 | 0.03 | 19.36 | 602.0 | 252.9 | 2.38 |
| 2013- RR0015 | LU sired | FOR | 498 | 690 | 7.31 | 0.03 | 28.99 | 666.5 | 184.2 | 3.62 |
| 2013- RR0016 | CH sired | FOR | 421 | 738 | 6.23 | **0.23** | 24.25 | 661.3 | 191.5 | 3.45 |
| 2013- RR0017 | LU sired | CONC | 469 | 694 | 11.91 | **0.44** | 12.71 | 604.0 | 270.7 | 2.23 |
| 2013- RR0018 | CH sired | CONC | 420 | 722 | 7.05 | 0.15 | 13.00 | 508.2 | 386.1 | 1.32 |
| 2013- RR0019 | CH sired | CONC | 488 | 766 | 10.29 | 0.04 | 14.69 | 654.0 | 179.4 | 3.65 |
| 2013- RR0020 | LU sired | CONC | 490 | 648 | 8.10 | 0.06 | 17.20 | 502.3 | 363.3 | 1.38 |
| 2013- RR0021 | CH sired | FOR | 427 | 678 | 6.47 | 0.02 | 21.92 | 620.9 | 200.1 | 3.10 |
| 2013- RR0022 | CH sired | FOR | 444 | 706 | 7.15 | 0.06 | 22.54 | 636.4 | 242.8 | 2.62 |
| 2013- RR0023 | LU sired | FOR | 467 | 736 | 7.36 | 0.03 | 21.79 | 677.6 | 205.9 | 3.29 |
| 2013- RR0024 | LU sired | FOR | 476 | 736 | 9.09 | 0.03 | 18.60 | 660.9 | 179.9 | 3.67 |
| 2014 Experiment (Forage diet) | | | | | | | | | | |
| 2014- RR0025 | Lim. sired | FOR | 389 | 680 | 5.16 | 0.04 | 28.53 | 656.8 | 147.9 | 4.44 |
| 2014- RR0026 | Lim. sired | FOR | 362 | 668 | 5.50 | 0.06 | 26.59 | 667.6 | 155.3 | 4.30 |
| 2014- RR0027 | Lim. sired | FOR | 415 | 716 | 7.29 | 0.02 | 21.94 | 675.2 | 168.6 | 4.00 |
| 2014- RR0028 | AA sired | FOR | 416 | 690 | 6.22 | 0.03 | 22.17 | 686.0 | 171.4 | 4.00 |
| 2014- RR0029 | AA sired | FOR | 426 | 622 | 6.39 | 0.03 | 24.81 | 655.0 | 144.0 | 4.55 |
| 2014- RR0030 | Lim. sired | FOR | 422 | 706 | 6.42 | 0.08 | 22.91 | 652.1 | 170.2 | 3.83 |
| 2014- RR0031 | AA sired | FOR | 380 | 686 | 6.45 | 0.06 | 17.51 | 647.9 | 176.6 | 3.67 |
| 2014- RR0032 | AA sired | FOR | 420 | 698 | 6.54 | 0.03 | 21.33 | 646.2 | 216.1 | 2.99 |
| 2014- RR0033 | AA sired | FOR | 378 | 684 | 7.29 | 0.01 | 21.94 | 655.9 | 205.5 | 3.19 |
| 2014- RR0034 | AA sired | FOR | 423 | 686 | 7.71 | 0.02 | 22.36 | 680.6 | 170.8 | 3.98 |
| 2014- RR0035 | Lim. sired | FOR | 418 | 668 | 7.85 | 0.05 | 22.10 | 684.9 | 188.5 | 3.63 |
| 2014- RR0036 | AA sired | FOR | 417 | 706 | 8.31 | 0.06 | 26.42 | 673.8 | 164.7 | 4.09 |
| 2014- RR0037 | Lim. sired | FOR | 417 | 684 | 8.80 | 0.09 | 21.64 | 671.4 | 168.4 | 3.99 |
| 2014- RR0038 | AA sired | FOR | 422 | 814 | 8.85 | 0.02 | 27.83 | 651.0 | 187.0 | 3.48 |
| 2014- RR0039 | Lim. sired | FOR | 411 | 722 | 8.86 | 0.05 | 18.65 | 658.2 | 186.9 | 3.52 |
| 2014- RR0040 | AA sired | FOR | 411 | 726 | 6.86 | 0.05 | 18.04 | 662.8 | 165.8 | 4.00 |
| 2014- RR0041 | Lim. Sired | FOR | 402 | 622 | 8.48 | 0.01 | 25.03 | N.D. | N.D. | N.D. |
| 2014- RR0042 | Lim. sired | FOR | 418 | 678 | 7.70 | 0.09 | 24.53 | 678.9 | 184.9 | 3.67 |

AA: Aberdeen Angus, Lim: Limousin, CH: Charolais, LU: Luing. FOR: Forage diet, CONC: Concentrate diet.

^1^ Possible rumen dysbiosis for values above 0.19 (in bold).

Table S2. Groups of AMR genes significantly correlated with abundance of the *Proteobacteria* phylum and *Proteobacteria* ratio.

| ***Proteobacteria* phylum/ratio** | **Description** | **Spearman’s correlation** |
| --- | --- | --- |
| *Proteobacteria* phylum | Aminoglycoside resistance | N.S. |
|  | Beta-lactam resistance | 0.261° |
|  | Chloramphenicol resistance | -0.269° |
|  | Macrolide resistance | N.S. |
|  | Microcin resistance | N.S. |
|  | Streptomycin resistance | N.S. |
|  | Fosmidomycin resistance | N.S. |
| *Proteobacteria* ratio | Aminoglycoside resistance | N.S. |
|  | Beta-lactam resistance | 0.579^**^ |
|  | Chloramphenicol resistance | -0.484^**^ |
|  | Macrolide resistance | 0.243° |
|  | Microcin resistance | -0.260 ° |
|  | Streptomycin resistance | N.S. |
|  | Fosmidomycin resistance | N.S. |

Sum of gene abundance (value > 0.001 %)

** *P*<0.01, **P*<0.05, °*P*<0.1, NS: not significant.

Table S3. The relative abundance of AMR genes.

| **KEGG genes database** ^1^ | | | | |
| --- | --- | --- | --- | --- |
| Gene | Function | Mean Forage | Mean Concentrate | *P-*Value |
| K00638 | chloramphenicol O-acetyltransferase | 0.0112 | 0.0021 | 0.030 |
| K00662 | aminoglycoside N3prime-acetyltransferase | 0.0111 | 0.0005 | 0.011 |
| K00897 | aminoglycoside 3prime-phosphotransferase | 0.0004 | 0.0000 | 0.023 |
| K00984 | streptomycin 3prime-adenylyltransferase | 0.0145 | 0.0007 | 0.003 |
| K01467 | beta-lactamase | 0.0042 | 0.0005 | 0.003 |
| K02547 | methicillin resistance protein | 0.0148 | 0.0146 | 0.938 |
| K04343 | streptomycin 6-kinase | 0.0000 | 0.0004 | 0.001 |
| K05593 | aminoglycoside 6-adenylyltransferase | 0.0643 | 0.1767 | 0.000 |
| K05595 | multiple antibiotic resistance protein | 0.0005 | 0.0010 | 0.005 |
| K07552 | MFS transporter, DHA1 family, bicyclomycin/chloramphenicol resistance protein | 0.0000 | 0.0004 | 0.002 |
| K07576 | metallo-beta-lactamase family protein | 0.0845 | 0.0256 | 0.000 |
| K08164 | MFS transporter, DHA1 family, chloramphenicol resistance protein | 0.0003 | 0.0005 | 0.198 |
| K08217 | MFS transporter, DHA3 family, macrolide efflux protein | 0.0009 | 0.0062 | 0.000 |
| K08218 | MFS transporter, PAT family, beta-lactamase induction signal transducer AmpG | 0.0021 | 0.0095 | 0.000 |
| K08223 | MFS transporter, FSR family, fosmidomycin resistance protein | 0.0001 | 0.0001 | 0.322 |
| K10673 | streptomycin 3prime-kinase | 0.0064 | 0.0015 | 0.002 |
| K13632 | AraC family transcriptional regulator, multiple antibiotic resistance protein MarA | 0.0001 | 0.0001 | 0.644 |
| K13893 | microcin C transport system substrate-binding protein | 0.0000 | 0.0000 | 0.191 |
| K13894 | microcin C transport system permease protein | 0.0000 | 0.0000 | 0.004 |
| K13895 | microcin C transport system permease protein | 0.0000 | 0.0000 | 0.161 |
| K13896 | microcin C transport system ATP-binding protein | 0.0001 | 0.0001 | 0.582 |
| **SRST2 genes database** ^1^ | | | | |
| Gene | Function | Mean Forage | Mean Concentrate | *P-*Value |
| *ant6-Ia* | Aminoglycoside nucleotidyltransferase gene | 0.0000 | 0.0017 | 0.0060 |
| *sat4A* | Aminoglycoside resitance/ streptothricin acetyltransferases | 0.0000 | 0.0001 | 0.3437 |
| *aph3-III* | Aminoglycoside-3'-phosphotransferase | 0.0000 | 0.0012 | 0.0087 |
| *ant6-Ib* | Aminoglycoside nucleotidyltransferase gene/ streptomycin resistance genes | 0.0001 | 0.0002 | 0.2444 |
| *mefA* | Macrolide resistance efflux pump | 0.0035 | 0.0018 | 0.0527 |
| *ermB* | macrolide-lincosamide-streptogramin rRNA adenine N-6-methyltransferase | 0.0000 | 0.0002 | 0.3230 |
| *ermF* | macrolide-lincosamide-streptogramin rRNA adenine N-6-methyltransferase | 0.0002 | 0.1209 | 0.0000 |
| *ermG* | macrolide-lincosamide-streptogramin rRNA adenine N-6-methyltransferase | 0.0000 | 0.0000 | 0.3332 |
| *lnuC* | macrolide-lincosamide-streptogramin transposon-mediated lincosamide nucleotidyltransferase | 0.0019 | 0.0076 | 0.0000 |
| *sulII* | sulfonamide resistance | 0.0005 | 0.0001 | 0.1588 |
| *tet(40)* | Tetracycline Efflux Gene | 0.0009 | 0.0007 | 0.5430 |
| *tetA-P* | Tetracycline Efflux Gene | 0.0001 | 0.0163 | 0.0599 |
| *tet(X)* | Tetracycline modification proteins | 0.0000 | 0.0001 | 0.0977 |
| *tet(32)* | Tetracycline Ribosomal protection proteins | 0.0005 | 0.0115 | 0.0549 |
| *tet(44)* | Tetracycline Ribosomal protection proteins | 0.0058 | 0.0013 | 0.0000 |
| *tet(O)* | Tetracycline Ribosomal protection proteins | 0.0100 | 0.1638 | 0.0000 |
| *tet(Q)* | Tetracycline Ribosomal protection proteins | 0.0596 | 0.0031 | 0.0000 |
| *tet(W)* | Tetracycline Ribosomal protection proteins | 0.1034 | 0.0106 | 0.0000 |
| *aCI-1* | β-Lactamase | 0.0011 | 0.0002 | 0.0000 |
| *cfxA* | β-Lactamase | 0.0000 | 0.0015 | 0.0101 |

^1^ No selection on the relative abundance of AMR genes.

Table S4: *Proteobacteria* populations strongly correlated with the *Proteobacteria* ratio

| **Bacterial Family** ^1^ | **Forage** | **Concentrate** | **All** | **Spearman’s correlation with *Proteobacteria* ratio^b^** |
| --- | --- | --- | --- | --- |
| *Proteobacteria* (Phylum) | 5.689 | 8.033 * | 6.360 | 0.919 |
| *Moraxellaceae* **^a^** | 0.046 | 0.069 ** | 0.044 | 0.949 |
| *Aeromonadaceae* **^a^** | 0.034 | 0.278 ** | 0.131 | 0.932 |
| *Pseudoalteromonadaceae* **^a^** | 0.072 | 0.017 ** | 0.011 | 0.931 |
| *Orbaceae* | 0.009 | 0.027 ** | 0.011 | 0.920 |
| *Wenzhouxiangellaceae* | 0.004 | 0.025 ** | 0.010 | 0.912 |
| *Hafniaceae* **^a^** | 0.003 | 0.052 ** | 0.030 | 0.912 |
| *Thiotrichaceae* | 0.020 | 0.007 ** | 0.003 | 0.912 |
| *Gallionellaceae* | 0.001 | 0.018 * | 0.010 | 0.911 |
| *Microbulbiferaceae* | 0.009 | 0.030 ** | 0.015 | 0.905 |
| *Oceanospirillaceae* | 0.009 | 0.020 ** | 0.013 | 0.901 |
| *Immundisolibacteraceae* | 0.010 | 0.021 * | 0.012 | 0.899 |
| *Nitrosomonadaceae* | 0.009 | 0.028 ** | 0.015 | 0.895 |
| *Erwiniaceae* | 0.009 | 0.111 ** | 0.070 | 0.888 |
| *Alcanivoracaceae* | 0.054 | 0.027 ** | 0.016 | 0.885 |
| *Halomonadaceae* | 0.011 | 0.077 ** | 0.050 | 0.875 |
| *Colwelliaceae* | 0.039 | 0.008 * | 0.005 | 0.874 |
| *Enterobacteriaceae* **^a^** | 0.001 | 0.374 * | 0.265 | 0.872 |
| *Ferrimonadaceae* | 0.223 | 0.012 ° | 0.008 | 0.866 |
| *Vibrionaceae* **^a^** | 0.007 | 0.068 ° | 0.055 | 0.851 |
| *Yersiniaceae* **^a^** | 0.049 | 0.090 ° | 0.072 | 0.845 |
| **Bacterial Genus** ^1^ | **Forage** | **Concentrate** | **All** | **Spearman’s correlation with *Proteobacteria* ratio^b^** |
| *Tolumonas* | 0.006 | 0.037 ** | 0.015 | 0.974 |
| *Moraxella* **^a^** | 0.006 | 0.028 ** | 0.013 | 0.968 |
| *Histophilus* **^a^** | 0.005 | 0.025 ** | 0.011 | 0.965 |
| *Grimontia* **^a^** | 0.003 | 0.010 ** | 0.005 | 0.952 |
| *Candidatus Ishikawaella* | 0.001 | 0.013 ** | 0.004 | 0.933 |
| *Pseudoalteromonas* | 0.009 | 0.018 ** | 0.012 | 0.930 |
| *Nitrosospira* | 0.007 | 0.006 | 0.007 | 0.928 |
| *Thioploca* | 0.001 | 0.007 ** | 0.002 | 0.924 |
| *Sideroxydans* | 0.005 | 0.018 ** | 0.009 | 0.922 |
| *Aeromonas* **^a^** | 0.065 | 0.253 ** | 0.119 | 0.921 |
| *Erwinia* | 0.014 | 0.028 ** | 0.018 | 0.918 |
| *Frischella* | 0.002 | 0.025 ** | 0.009 | 0.917 |
| *Hafnia* **^a^** | 0.002 | 0.006 * | 0.003 | 0.914 |
| *Wenzhouxiangella* | 0.005 | 0.026 ** | 0.011 | 0.913 |
| *Sodalis* | 0.009 | 0.025 ** | 0.014 | 0.911 |
| *Actinobacillus* **^a^** | 0.007 | 0.015 * | 0.009 | 0.909 |
| *Microbulbifer* | 0.010 | 0.033 ** | 0.017 | 0.902 |
| *Pantoea* **^a^** | 0.024 | 0.061 ** | 0.034 | 0.898 |
| *Methylomonas* | 0.011 | 0.025 ** | 0.015 | 0.897 |
| *Halomonas* | 0.027 | 0.058 * | 0.036 | 0.894 |

^1^ Population abundance (value > 0.001 %); ** *P*<0.01, **P*<0.05, °*P*<0.1. ^a^ Pathogens, **^b^** (*P*<0.001).

Table S5. Functional genes significantly correlated with *Proteobacteria* ratio (PLS).

| **KEGG ID^1^** | **Gene function** | **VIP** | **Coefficient** | **Cluster 2011** | **Cluster 2013** | **Cluster 2014** |
| --- | --- | --- | --- | --- | --- | --- |
| K00638 | chloramphenicol O-acetyltransferase | 0.971 | -0.050 | 6 | N.D. | N.D. |
| K00662 | aminoglycoside N3prime-acetyltransferase | 0.847 | 0.035 | N.D. | N.D. | N.D. |
| K01467 | beta-lactamase | 0.814 | 0.008 | 3 | 2 | 1 |
| K01839 | phosphopentomutase | 1.086 | 0.023 | 1 | 9 | N.D. |
| K02005 | HlyD family secretion protein | 0.911 | 0.061 | N.D. | N.D. | 14 |
| K02217 | ferritin | 0.937 | -0.055 | 3 | 1 | 3 |
| K02392 | flagellar basal-body rod protein FlgG | 1.128 | 0.031 | 1 | 6 | 1 |
| K02393 | flagellar L-ring protein precursor FlgH | 0.855 | 0.057 | N.D. | N.D. | N.D. |
| K02394 | flagellar P-ring protein precursor FlgI | 1.727 | 0.106 | 1 | N.D. | N.D. |
| K02395 | flagellar protein FlgJ | 1.288 | 0.055 | 1 | 6 | N.D. |
| K02396 | flagellar hook-associated protein 1 FlgK | 0.959 | 0.016 | 48 | 6 | N.D. |
| K02398 | negative regulator of flagellin synthesis FlgM | 1.086 | 0.007 | 1 | 9 | N.D. |
| K02400 | flagellar biosynthesis protein FlhA | 1.279 | 0.050 | 1 | N.D. | N.D. |
| K02406 | flagellin | 1.122 | 0.027 | 1 | 6 | N.D. |
| K02407 | flagellar hook-associated protein 2 | 1.060 | 0.002 | 1 | 6 | N.D. |
| K02412 | flagellum-specific ATP synthase | 1.041 | -0.021 | 1 | N.D. | N.D. |
| K02417 | flagellar motor switch protein FliN/FliY | 0.895 | -0.055 | N.D. | N.D. | N.D. |
| K02419 | flagellar biosynthetic protein FliP | 0.907 | -0.011 | 8 | N.D. | N.D. |
| K02422 | flagellar protein FliS | 1.073 | -0.035 | 1 | N.D. | N.D. |
| K02429 | MFS transporter, FHS family, L-fucose permease | 0.825 | 0.053 | 28 | 18 | N.D. |
| K02461 | general secretion pathway protein L | 1.072 | -0.037 | 1 | N.D. | N.D. |
| K02656 | type IV pilus assembly protein PilF | 1.211 | 0.080 | N.D. | N.D. | N.D. |
| K02843 | heptosyltransferase II | 0.815 | 0.019 | 3 | 2 | N.D. |
| K03274 | ADP-L-glycero-D-manno-heptose 6-epimerase | 1.104 | -0.012 | 1 | 2 | N.D. |
| K03564 | peroxiredoxin Q/BCP | 1.165 | 0.071 | 1 | N.D. | N.D. |
| K03704 | cold shock protein (beta-ribbon, CspA family) | 1.015 | -0.019 | 1 | 24 | N.D. |
| K04061 | flagellar biosynthesis protein | 1.145 | 0.034 | 1 | 6 | N.D. |
| K06603 | flagellar protein FlaG | 1.207 | 0.061 | 1 | 6 | N.D. |
| K07576 | metallo-beta-lactamase family protein | 1.227 | 0.036 | 1 | 9 | N.D. |
| K07638 | two-component system, OmpR family, osmolarity sensor histidine kinase EnvZ | 1.013 | 0.067 | N.D. | N.D. | N.D. |
| K07642 | two-component system, OmpR family, sensor histidine kinase BaeS | 0.903 | 0.022 | 3 | 2 | N.D. |
| K07645 | two-component system, OmpR family, sensor histidine kinase QseC | 0.856 | 0.057 | N.D. | N.D. | N.D. |
| K07657 | two-component system, OmpR family, phosphate regulon response regulator PhoB | 0.982 | 0.026 | 1 | N.D. | N.D. |
| K07665 | two-component system, OmpR family, copper resistance phosphate regulon response regulator CusR | 0.850 | 0.015 | 3 | 2 | N.D. |
| K07678 | two-component system, NarL family, sensor histidine kinase BarA | 0.925 | 0.062 | N.D. | N.D. | N.D. |
| K07718 | two-component system, sensor histidine kinase YesM | 1.080 | 0.026 | 1 | 6 | N.D. |
| K08161 | MFS transporter, DHA1 family, multidrug resistance protein | 0.913 | 0.061 | N.D. | N.D. | N.D. |
| K11065 | thiol peroxidase, atypical 2-Cys peroxiredoxin | 0.939 | 0.021 | 3 | 2 | N.D. |
| K11891 | type VI secretion system protein ImpL | 1.531 | 0.102 | N.D. | N.D. | N.D. |
| K11898 | type VI secretion system protein ImpE | 1.071 | -0.035 | N.D. | N.D. | N.D. |
| K11900 | type VI secretion system protein ImpC | 0.975 | -0.023 | 1 | N.D. | N.D. |
| K11907 | type VI secretion system protein VasG | 1.063 | 0.037 | 1 | N.D. | N.D. |
| K13626 | flagellar assembly factor FliW | 0.912 | 0.021 | 1 | N.D. | N.D. |

VIP: Variable Importance for Projection, N.D.: Not Detected.

^1^Gene abundance (value > 0.001 %)

** *P*<0.01, **P*<0.05, N.D.: Not Determined

Table S6. Cluster distribution of functional genes significantly different between diets.

| **KEGG ID^1^** | **Function** | ***P*-value** | **Cluster 2011** | **Cluster 2013** | **Cluster 2014** |
| --- | --- | --- | --- | --- | --- |
| **Genes correlated with Diet and *Proteobacteria* ratio** | | | | | |
| K00638 | chloramphenicol O-acetyltransferase | 0.00000 | 6 | N.D. | N.D. |
| K01467 | beta-lactamase | 0.00301 | 3 | 2 | 1 |
| K01839 | phosphopentomutase | 0.00045 | 1 | 9 | N.D. |
| K02217 | ferritin | 0.00000 | 3 | 1 | 3 |
| K02392 | flagellar basal-body rod protein FlgG | 0.04783 | 1 | 6 | 1 |
| K02394 | flagellar P-ring protein precursor FlgI | 0.01182 | 1 | N.D. | N.D. |
| K02395 | flagellar protein FlgJ | 0.01989 | 1 | 6 | N.D. |
| K02400 | flagellar biosynthesis protein FlhA | 0.00842 | 1 | N.D. | N.D. |
| K02406 | flagellin | 0.04315 | 1 | 6 | N.D. |
| K02407 | flagellar hook-associated protein 2 | 0.08770 | 1 | 6 | N.D. |
| K02412 | flagellum-specific ATP synthase | 0.04295 | 1 | N.D. | N.D. |
| K02417 | flagellar motor switch protein FliN/FliY | 0.00000 | N.D. | N.D. | N.D. |
| K02843 | heptosyltransferase II | 0.00214 | 3 | 2 | N.D. |
| K03274 | ADP-L-glycero-D-manno-heptose 6-epimerase | 0.00326 | 1 | 2 | N.D. |
| K03564 | peroxiredoxin Q/BCP | 0.03516 | 1 | N.D. | N.D. |
| K03704 | cold shock protein (beta-ribbon, CspA family) | 0.00042 | 1 | 24 | N.D. |
| K03781 | catalase | 0.02253 | N.D. | N.D. | 1 |
| K04061 | flagellar biosynthesis protein | 0.03069 | 1 | 6 | N.D. |
| K04758 | ferrous iron transport protein A | 0.03183 | 5 | N.D. | N.D. |
| K07576 | metallo-beta-lactamase family protein | 0.00305 | 1 | 9 | N.D. |
| K07642 | two-component system, OmpR family, sensor histidine kinase BaeS | 0.00109 | 3 | 2 | N.D. |
| K07657 | two-component system, OmpR family, phosphate regulon response regulator PhoB | 0.00111 | 1 | N.D. | N.D. |
| K07665 | two-component system, OmpR family, copper resistance phosphate regulon response regulator CusR | 0.00120 | 3 | 2 | N.D. |
| K11065 | thiol peroxidase, atypical 2-Cys peroxiredoxin | 0.00197 | 3 | 2 | N.D. |
| K11907 | type VI secretion system protein VasG | 0.01246 | 1 | N.D. | N.D. |
| **Genes only correlated with diet** | | | | | |
| K00432 | glutathione peroxidase | 0.00055 | N.D. | N.D. | N.D. |
| K00897 | aminoglycoside 3prime-phosphotransferase | 0.03030 | 11 | N.D. | N.D. |
| K00984 | streptomycin 3prime-adenylyltransferase | 0.00198 | N.D. | N.D. | N.D. |
| K01206 | alpha-L-fucosidase | 0.01469 | N.D. | 10 | N.D. |
| K01835 | phosphoglucomutase | 0.02248 | N.D. | 3 | 2 |
| K01993 | HlyD family secretion protein | 0.00808 | 15 | 5 | N.D. |
| K02016 | iron complex transport system substrate-binding protein | 0.00467 | N.D. | N.D. | N.D. |
| K02416 | flagellar motor switch protein FliM | 0.03541 | 44 | N.D. | N.D. |
| K02456 | general secretion pathway protein G | 0.00756 | 47 | 2 | N.D. |
| K02652 | type IV pilus assembly protein PilB | 0.03019 | 29 | N.D. | 13 |
| K02662 | type IV pilus assembly protein PilM | 0.00007 | 2 | 5 | 4 |
| K02663 | type IV pilus assembly protein PilN | 0.00002 | 2 | 5 | 4 |
| K02664 | type IV pilus assembly protein PilO | 0.00009 | 2 | 5 | 4 |
| K02666 | type IV pilus assembly protein PilQ | 0.00000 | 2 | 5 | 4 |
| K03297 | small multidrug resistance protein, SMR family | 0.00008 | 22 | N.D. | N.D. |
| K04564 | superoxide dismutase, Fe-Mn family | 0.03098 | N.D. | N.D. | N.D. |
| K04759 | ferrous iron transport protein B | 0.00233 | N.D. | N.D. | N.D. |
| K05593 | aminoglycoside 6-adenylyltransferase | 0.01111 | 34 | N.D. | 1 |
| K05685 | macrolide transport system ATP-binding/permease protein | 0.04903 | 1 | N.D. | N.D. |
| K07552 | MFS transporter, DHA1 family, bicyclomycin/chloramphenicol resistance protein | 0.00542 | N.D. | 4 | 1 |
| K07668 | two-component system, OmpR family, response regulator VicR | 0.02893 | 2 | N.D. | 4 |
| K08217 | MFS transporter, DHA3 family, macrolide efflux protein | 0.01203 | N.D. | N.D. | N.D. |
| K09686 | antibiotic transport system permease protein | 0.00032 | N.D. | N.D. | N.D. |
| K13894 | microcin C transport system permease protein | 0.00655 | 2 | 5 | 4 |
| K13895 | microcin C transport system permease protein | 0.00000 | 2 | 5 | 4 |
| K08217 | MFS transporter, DHA3 family, macrolide efflux protein | 0.00000 | N.D. | N.D. | N.D. |

^1^ Gene abundance (value > 0.001 %); N.D.: Not detected.
