# Supplementary figures and images for: Correction to: The rumen microbiome as a reservoir of antimicrobial resistance and pathogenicity genes is directly affected by diet in beef cattle
Source: Microbiome. 2019 Nov 18;7:149. doi: 10.1186/s40168-019-0764-9 (PMC6862725; doi:10.1186/s40168-019-0764-9)

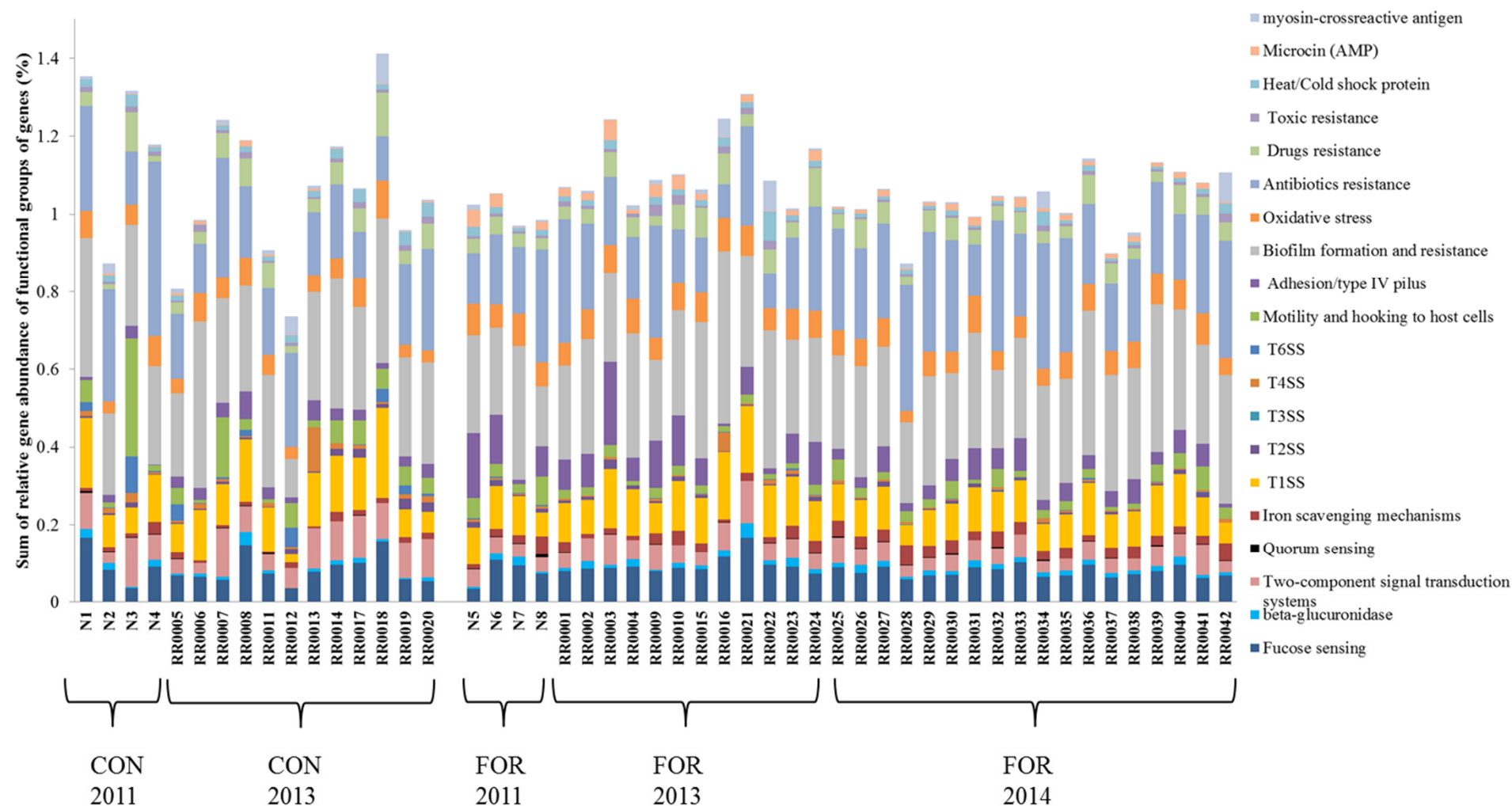

Supplement: Supplementary file 1 — Additional file 1: Figure S1.Relative abundance (%) of 20 groups of functional genes representing 204 selected genes (number of animals, n = 50 samples). The sum of the relative abundance (%) of genes grouping within the same function is shown in this figure. Figure S2A. Total abundance of 204 selected genes based on diet treatments (n = 50). *P value < 0.05. Figure S2B. Shannon index diversity of 204 selected genes based on diet treatments (n = 50). *P value < 0.05, °P value < 0.1. Figure S3. Canonical Variate analysis (CVA) on the structure of 204 genes selected based on breed, age, weight, Proteobacteria ratio, FCR and methane grouping (n = 50). Figure S4. Factors explaining the significant differences observed for Proteobacteria ratio (n = 50). Figure S5. Microbial community composition at the phylum level (n = 50). Table S1. Characteristics of the cattle used in the experiment. Table S2. Groups of AMR genes significantly correlated with abundance of the Proteobacteria phylum and Proteobacteria ratio. Table S3. The relative abundance of AMR genes. Table S4, Proteobacteria populations strongly correlated with the Proteobacteria ratio. Table S5. Functional genes significantly correlated with Proteobacteria ratio (PLS). Table S6. Cluster distribution of functional genes significantly different between diets. [file 40168_2019_764_MOESM1_ESM.zip › FigS1.pdf]

A.

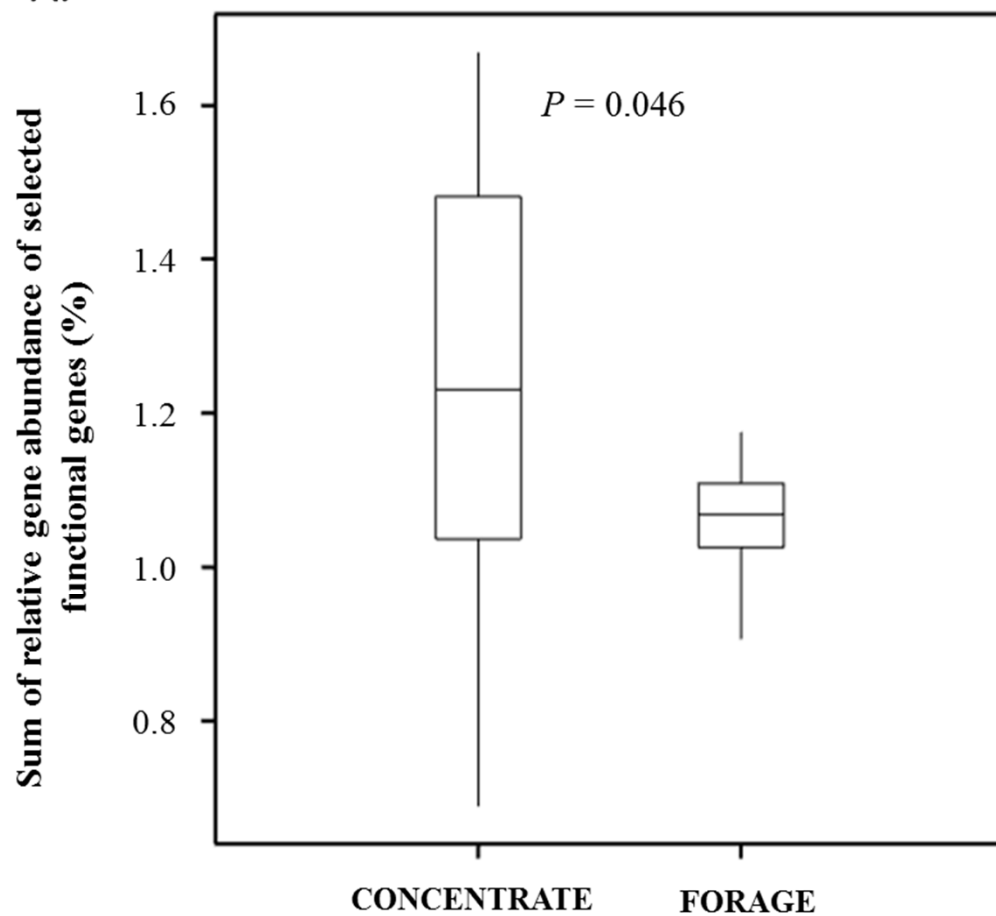

B.

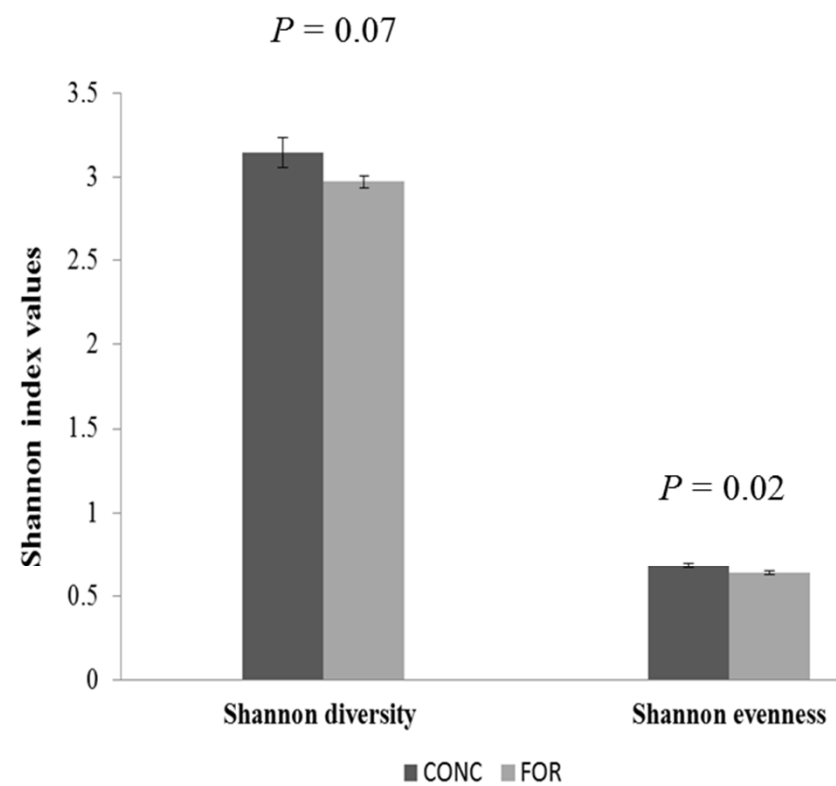

Supplement: Supplementary file 1 — Additional file 1: Figure S1.Relative abundance (%) of 20 groups of functional genes representing 204 selected genes (number of animals, n = 50 samples). The sum of the relative abundance (%) of genes grouping within the same function is shown in this figure. Figure S2A. Total abundance of 204 selected genes based on diet treatments (n = 50). *P value < 0.05. Figure S2B. Shannon index diversity of 204 selected genes based on diet treatments (n = 50). *P value < 0.05, °P value < 0.1. Figure S3. Canonical Variate analysis (CVA) on the structure of 204 genes selected based on breed, age, weight, Proteobacteria ratio, FCR and methane grouping (n = 50). Figure S4. Factors explaining the significant differences observed for Proteobacteria ratio (n = 50). Figure S5. Microbial community composition at the phylum level (n = 50). Table S1. Characteristics of the cattle used in the experiment. Table S2. Groups of AMR genes significantly correlated with abundance of the Proteobacteria phylum and Proteobacteria ratio. Table S3. The relative abundance of AMR genes. Table S4, Proteobacteria populations strongly correlated with the Proteobacteria ratio. Table S5. Functional genes significantly correlated with Proteobacteria ratio (PLS). Table S6. Cluster distribution of functional genes significantly different between diets. [file 40168_2019_764_MOESM1_ESM.zip › FigS2.pdf]

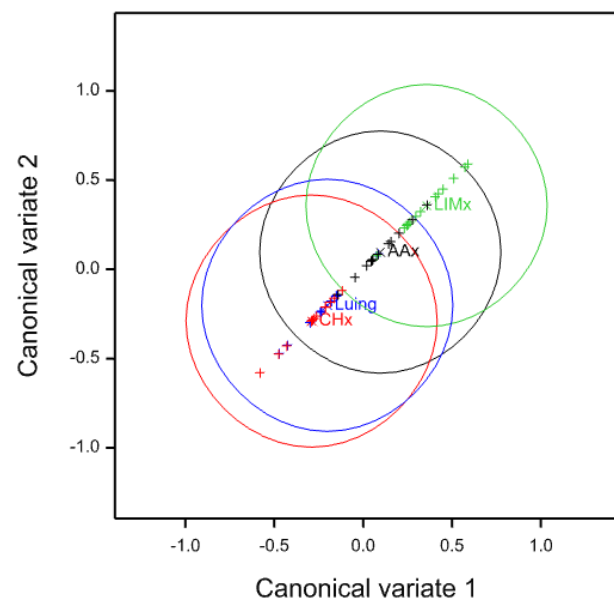

Breed

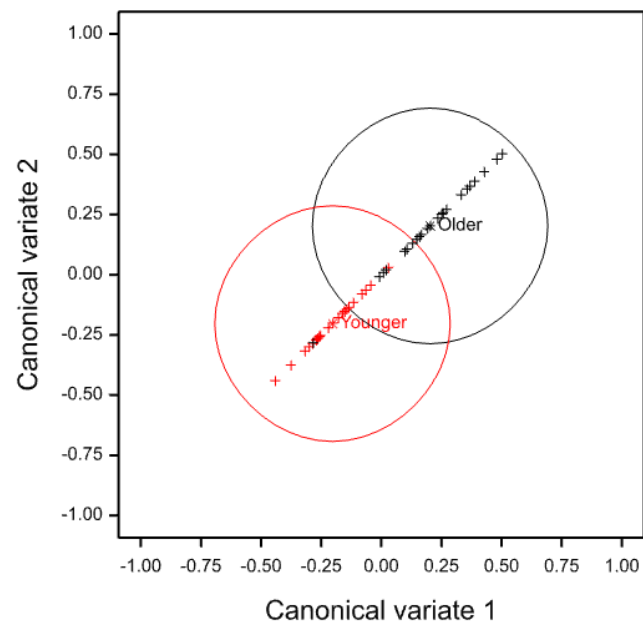

Age

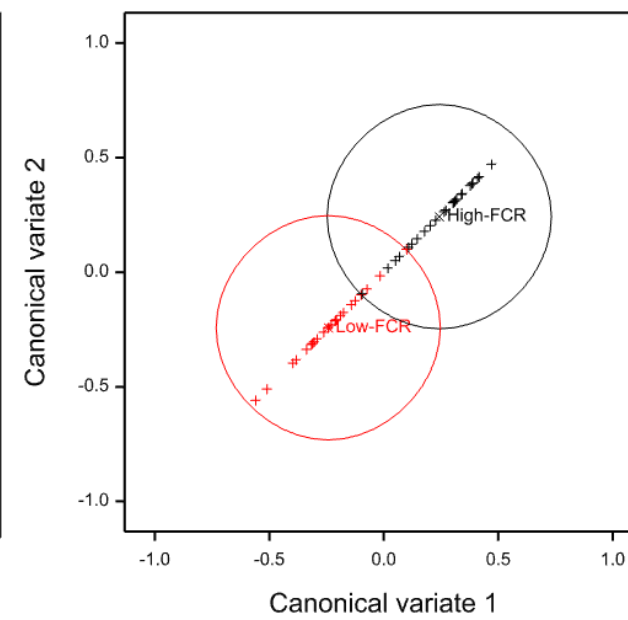

FCR

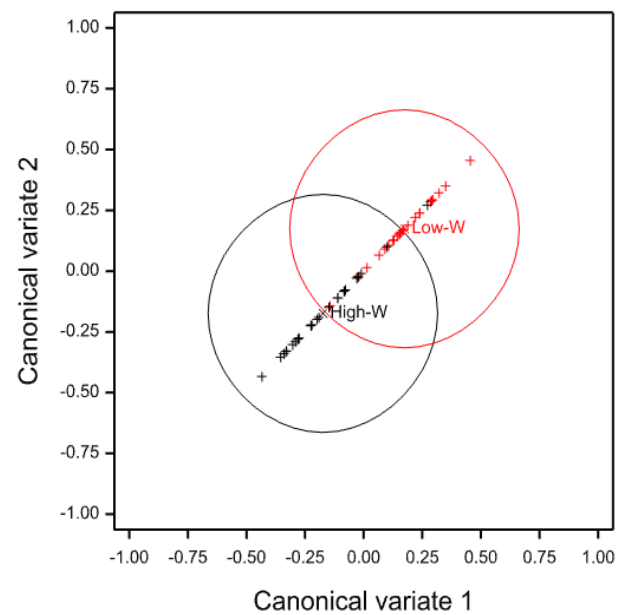

Weight

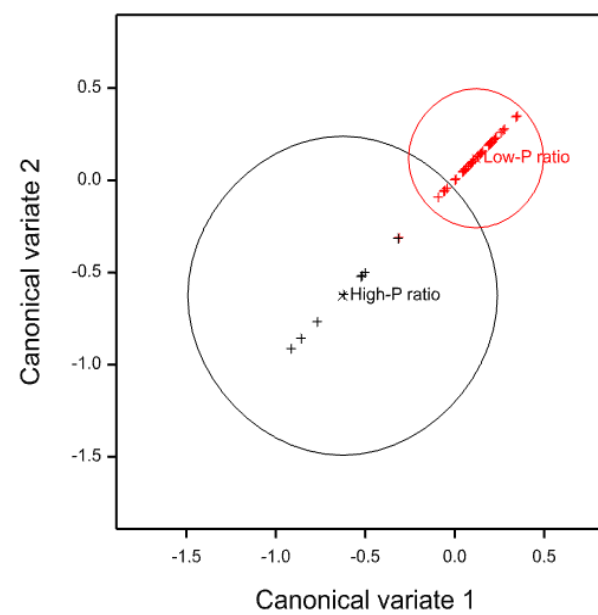

Proteobacteria ratio

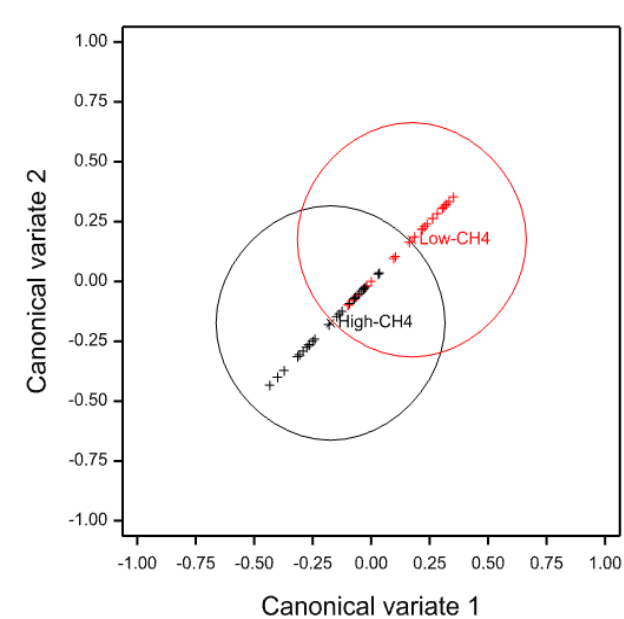

Methane

Supplement: Supplementary file 1 — Additional file 1: Figure S1.Relative abundance (%) of 20 groups of functional genes representing 204 selected genes (number of animals, n = 50 samples). The sum of the relative abundance (%) of genes grouping within the same function is shown in this figure. Figure S2A. Total abundance of 204 selected genes based on diet treatments (n = 50). *P value < 0.05. Figure S2B. Shannon index diversity of 204 selected genes based on diet treatments (n = 50). *P value < 0.05, °P value < 0.1. Figure S3. Canonical Variate analysis (CVA) on the structure of 204 genes selected based on breed, age, weight, Proteobacteria ratio, FCR and methane grouping (n = 50). Figure S4. Factors explaining the significant differences observed for Proteobacteria ratio (n = 50). Figure S5. Microbial community composition at the phylum level (n = 50). Table S1. Characteristics of the cattle used in the experiment. Table S2. Groups of AMR genes significantly correlated with abundance of the Proteobacteria phylum and Proteobacteria ratio. Table S3. The relative abundance of AMR genes. Table S4, Proteobacteria populations strongly correlated with the Proteobacteria ratio. Table S5. Functional genes significantly correlated with Proteobacteria ratio (PLS). Table S6. Cluster distribution of functional genes significantly different between diets. [file 40168_2019_764_MOESM1_ESM.zip › FigS3.pdf]

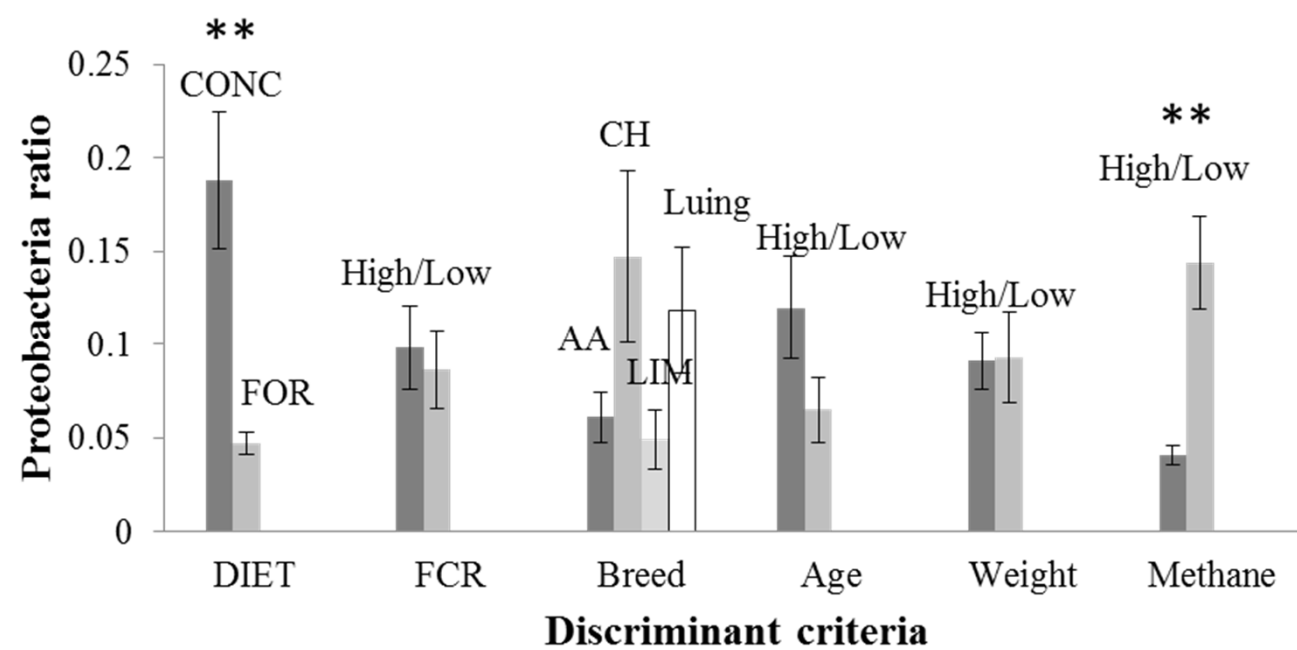

Supplement: Supplementary file 1 — Additional file 1: Figure S1.Relative abundance (%) of 20 groups of functional genes representing 204 selected genes (number of animals, n = 50 samples). The sum of the relative abundance (%) of genes grouping within the same function is shown in this figure. Figure S2A. Total abundance of 204 selected genes based on diet treatments (n = 50). *P value < 0.05. Figure S2B. Shannon index diversity of 204 selected genes based on diet treatments (n = 50). *P value < 0.05, °P value < 0.1. Figure S3. Canonical Variate analysis (CVA) on the structure of 204 genes selected based on breed, age, weight, Proteobacteria ratio, FCR and methane grouping (n = 50). Figure S4. Factors explaining the significant differences observed for Proteobacteria ratio (n = 50). Figure S5. Microbial community composition at the phylum level (n = 50). Table S1. Characteristics of the cattle used in the experiment. Table S2. Groups of AMR genes significantly correlated with abundance of the Proteobacteria phylum and Proteobacteria ratio. Table S3. The relative abundance of AMR genes. Table S4, Proteobacteria populations strongly correlated with the Proteobacteria ratio. Table S5. Functional genes significantly correlated with Proteobacteria ratio (PLS). Table S6. Cluster distribution of functional genes significantly different between diets. [file 40168_2019_764_MOESM1_ESM.zip › FigS4.pdf]

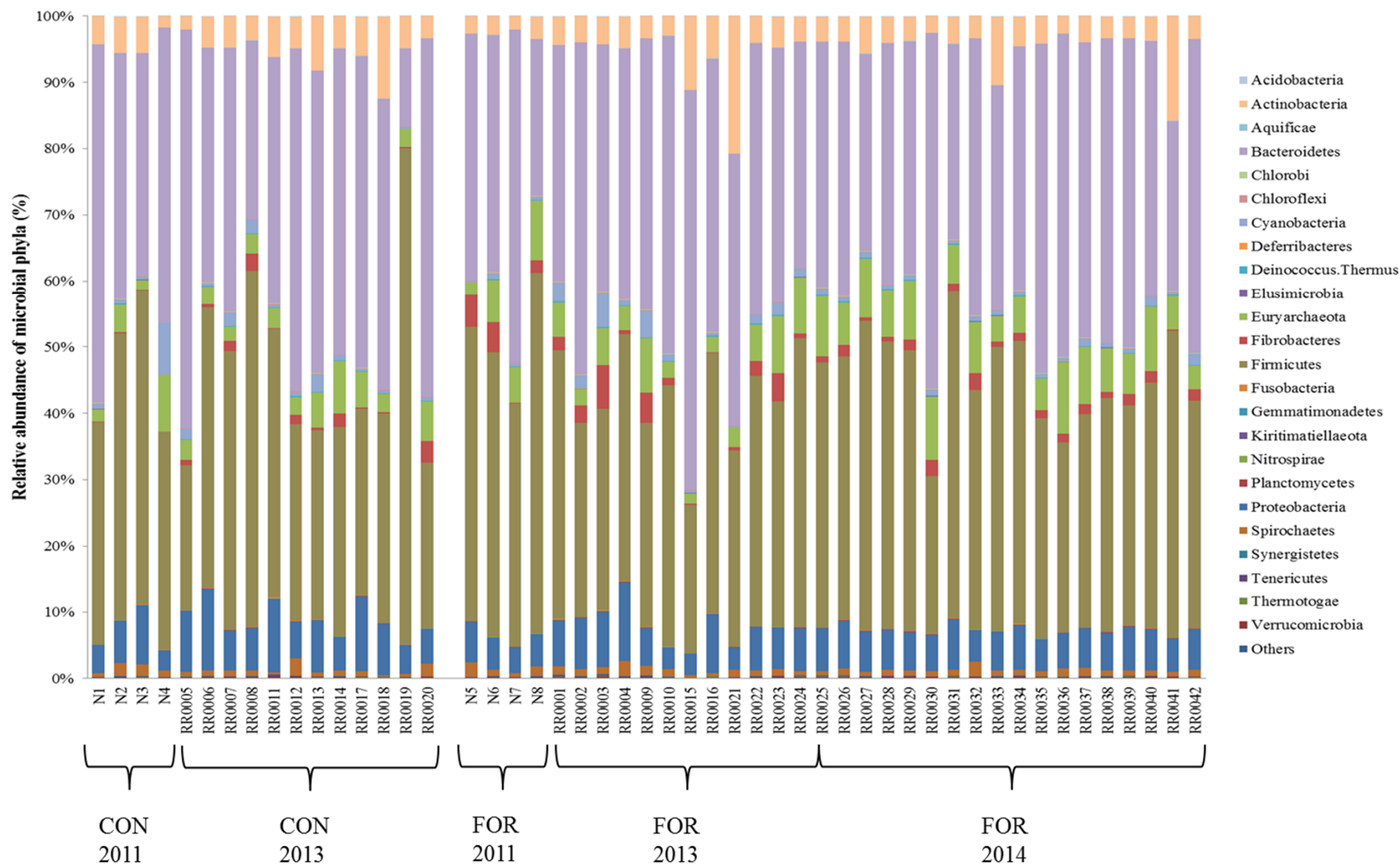

Supplement: Supplementary file 1 — Additional file 1: Figure S1.Relative abundance (%) of 20 groups of functional genes representing 204 selected genes (number of animals, n = 50 samples). The sum of the relative abundance (%) of genes grouping within the same function is shown in this figure. Figure S2A. Total abundance of 204 selected genes based on diet treatments (n = 50). *P value < 0.05. Figure S2B. Shannon index diversity of 204 selected genes based on diet treatments (n = 50). *P value < 0.05, °P value < 0.1. Figure S3. Canonical Variate analysis (CVA) on the structure of 204 genes selected based on breed, age, weight, Proteobacteria ratio, FCR and methane grouping (n = 50). Figure S4. Factors explaining the significant differences observed for Proteobacteria ratio (n = 50). Figure S5. Microbial community composition at the phylum level (n = 50). Table S1. Characteristics of the cattle used in the experiment. Table S2. Groups of AMR genes significantly correlated with abundance of the Proteobacteria phylum and Proteobacteria ratio. Table S3. The relative abundance of AMR genes. Table S4, Proteobacteria populations strongly correlated with the Proteobacteria ratio. Table S5. Functional genes significantly correlated with Proteobacteria ratio (PLS). Table S6. Cluster distribution of functional genes significantly different between diets. [file 40168_2019_764_MOESM1_ESM.zip › FigS5.pdf]
